# Procyanidin—Cell Wall Interactions within Apple Matrices Decrease the Metabolization of Procyanidins by the Human Gut Microbiota and the Anti-Inflammatory Effect of the Resulting Microbial Metabolome In Vitro

**DOI:** 10.3390/nu11030664

**Published:** 2019-03-19

**Authors:** Carine Le Bourvellec, Priscilla Bagano Vilas Boas, Pascale Lepercq, Sophie Comtet-Marre, Pauline Auffret, Philippe Ruiz, Romain Bott, Catherine M. G. C. Renard, Claire Dufour, Jean-Marc Chatel, Pascale Mosoni

**Affiliations:** 1 UMR408 SQPOV «Sécurité et Qualité des Produits d’Origine Végétale», INRA, Avignon Université, F-84000 Avignon, France; carine.le-bourvellec@inra.fr (C.L.B.); romain.bott@inra.fr (R.B.); catherine.renard@inra.fr (C.M.G.C.R.); claire.dufour@inra.fr (C.D.); 2 Micalis, INRA, AgroParisTech, Université Paris-Saclay, F-78000 Jouy-en-Josas, France; bagano.vilasboas@gmail.com (P.B.V.B.); jean-marc.chatel@inra.fr (J.-M.C.); 3 Université Clermont Auvergne, INRA, UMR 0454 MEDIS, F-63000 Clermont-Ferrand, France; lepercq@insa-toulouse.fr (P.L.); sophie.marre@uca.fr (S.C.-M.); paulineauffret88@gmail.com (P.A.); philippe.ruiz@inra.fr (P.R.); 4 Ifremer, UMR 241 EIO, F-98702 Tahiti, French Polynesia

**Keywords:** in vitro batch fermentation, polyphenols, dietary fiber, 16S metabarcoding, metabotype

## Abstract

B-type oligomeric procyanidins in apples constitute an important source of polyphenols in the human diet. Their role in health is not known, although it is suggested that they generate beneficial bioactive compounds upon metabolization by the gut microbiota. During apple processing, procyanidins interact with cell-wall polysaccharides and form stable complexes. These interactions need to be taken into consideration in order to better assess the biological effects of fruit constituents. Our objectives were to evaluate the impact of these interactions on the microbial metabolization of cell walls and procyanidins, and to investigate the potential anti-inflammatory activity of the resulting metabolome, in addition to analyzing the taxonomical changes which the microbiota undergo. In vitro fermentation of three model apple matrices with microbiota from 4 healthy donors showed that the binding of procyanidins to cell-wall polysaccharides, whether covalently or non-covalently, substantially reduced procyanidin degradation. Although cell wall-unbound procyanidins negatively affected carbohydrate fermentation, they generated more hydroxyphenylvaleric acid than bound procyanidins, and increased the abundance of *Adlercreutzia* and *Gordonibacter* genera. The best results in terms of production of anti-inflammatory bioactive metabolites were observed from the apple matrix with no bonds between procyanidins and cell wall polysaccharides, although the matrix with non-covalent bonds was not far behind.

## 1. Introduction 

Dietary phenolic compounds are ubiquitous secondary metabolites that are abundant in fruits and vegetables. In apples, the most abundant phenolic compounds are procyanidins and hydroxycinnamic acids. B-type procyanidins, also known as condensed tannins, are oligomers and polymers mainly composed of (−)-epicatechin units, although some of the terminal units may also be (+)-catechin [1,2]. 

It is now accepted that the chronic consumption of fruits exerts anti-inflammatory effects in humans, but the underlying mechanisms are far from being understood [3]. Growing evidence indicates that the biological (anti-inflammatory, anti-oxidant etc.) effects of fruit non-extractable polyphenols (ellagitannins, flavanones, procyanidins) in humans could be attributed to their gut microbial metabolites rather than to native macromolecules [4]. Nonetheless, to our knowledge, the biological effect of gut microbial metabolites generated from apple procyanidins, and more generally of apple polyphenols, still needs to be demonstrated. 

In addition to our limited knowledge of procyanidin metabolization during digestion [5], we also need to take into account the fact that prior to ingestion, procyanidins can complex with other plant macromolecules [6]. It is now well established that during mechanical food processing or mastication, procyanidins released from plant cell vacuoles bind spontaneously via weak bonds (hydrogen and hydrophobic) to the extracellular cell wall polymers (cellulose, hemicelluloses, pectins, etc.), thus forming non-covalent complexes [7]. Moreover, apple processing involving thermal treatment in relatively acidic conditions, e.g., in the production of apple sauce, may lead to procyanidin depolymerization, generating carbocations which could then react with nucleophilic groups of the cell wall, hence forming covalent bonds [8]. Consequently, the formation of non-covalent and covalent bonds between procyanidins and cell wall polysaccharides before ingestion could subsequently modify the nutritional qualities of apple, in particular by modifying procyanidin and polysaccharide bioaccessibilities in the gut, but also by affecting their metabolization by the gut microbiota.

Procyanidins (free or cell wall-associated) are poorly bioavailable in the upper gut [9], and reach the colon where they become fermentable substrates for the commensal microbiota. Although the microbial catabolism of procyanidins is far from being thoroughly described, readily absorbable low-molecular weight metabolites have been identified and can be used as markers of procyanidin metabolization [10,11,12]. Several studies using human fecal microbiota and batch fermentation showed that the conversion rate of procyanidins into known microbial metabolites was much lower with purified procyanidins than with procyanidins either kept within the fruit matrix [11,13]) or mixed with fruit polysaccharides [14,15]. These studies mainly showed that providing carbohydrate nutrients as an energy source to the microbiota stimulated the metabolization of procyanidins, compared with procyanidins alone. Nevertheless, in these previous studies, the design of the apple matrices was not conceived to investigate the impact of linkages between procyanidin and cell wall polysaccharides on the microbial metabolization of both moieties. 

The first objective of this study was to investigate in vitro the effect of different types of interactions between procyanidins and plant cell walls within apple food matrices on the metabolism of cell wall polysaccharides and procyanidins by the human gut microbiota in order to determine the extent to which these interactions modulate the production of microbial metabolites. Hence, apple matrices similar in their composition, although differing in the type of linkages between procyanidins and cell walls, were specifically produced.

The second objective was to evaluate the potential beneficial impact of these apple food matrices on human health by determining whether the resulting microbial metabolites exerted anti-inflammatory activity on intestinal cells in vitro and also by assessing their prebiotic effect through the analysis of the microbiota composition.

## 2. Material and Methods

### 2.1. Standards and Chemicals

Acetonitrile of HPLC grade, acetic acid and *N*,*O*-bis(trimethylsilyl)trifluoroacetamide + 10% trimethylchlorosilane (BSTFA + 10% TMCS) were from Fischer Scientific (Pittsburgh, PA, USA). 5-*O*-caffeoylquinic acid, (+)-catechin, (−)-epicatechin, sucrose, glucose, fructose, citric acid, NaBH_4_, *N*-methylimidazole, acetic anhydride, benzoic acid, 3-hydroxybenzoic acid, 4-hydroxybenzoic acid, 3,4-dihydroxybenzoic acid, 3-phenylpropionic acid, phenylacetic acid, 3-hydroxyphenylacetic acid, 3-(4-hydroxyphenyl)propionic acid, dl-3-phenyllactic acid, dl-3-(4-hydroxyphenyl)lactic acid, 5-phenylvaleric acid, 3,4-dihydroxyphenylacetic acid and 2,4,5-trimethoxycinnamic acid and toluene-α−thiol were obtained from Sigma-Aldrich (Saint Quentin Fallavier, France). Phloretin, *p*-coumaric acid, quercetin and cyanidin-3-*O*-galactoside were obtained from Extrasynthese (Lyon, France). Phloridzin was obtained from Fluka (Buchs, Switzerland). Malic acid was obtained from R-Biopharm (Darmstadt, Germany). D3-Methanol was from Acros Organics (Geel, Belgium). Acetonitrile was analytical grade and from Fisher Scientific (Fair Lawn, NJ, USA). Ethyl acetate, dichloromethane and hexane obtained from VWR International (Radnor, PA, USA).

### 2.2. Preparation of Apple Matrices with Different Polyphenol-Cell Wall Linkages

#### 2.2.1. Plant Material

Apple fruits (Malus domestica Borkh.) of the Bedan cultivar at technological maturity were obtained from the experimental orchard of Institut Français des Productions Cidricoles (IFPC, Sées, France) in December, 2013. Organically produced cultivar Reinette de Flandre was from Bio-verger (Mrs Christine Boutin, Ambricourt, France) in October 2013. 

Whole apple from each cultivar without stalks were separately cut into pieces, frozen, freeze-dried and ground until utilization. Freeze-dried apple powders were used for extraction and purification of phenolic compounds and cell wall material (see Section 2.2.4 and Section 2.2.5).

#### 2.2.2. Industrial Processing of Apple Purée

In order to obtain non-covalent and covalent bonds between procyanidins and cell walls, two industrial processing were applied: a classical cold break treatment and a cold break treatment in acidic conditions with a higher pasteurization time. Apple purée processing was carried out by the Centre Technique de la Conservation des Produits Agricoles (CTCPA, Avignon, France). 

Stalks from 9 kg of fruits from Bedan and 9 kg of fruits from Reinette de Flandre were removed, and then fruits were washed and drained for 5 min. Apples were placed in a 44 L-Stephan (ProXES Hameln, Germany) and 250 mL of an ascorbic acid solution at 216 g/L were added. Fruits were crushed and heated to 95 °C in 6 min, and the temperature was kept at 95 °C for 1 min. Purée was then crushed using a colloidal blender (Fryma, Rheinfelden, Switzerland) and then transferred back in the Stephan before deaeration under vacuum (−0.85 b) with stirring for 3 min. Apple purée was hot conditioned in 80 g-cans and pasteurized at 100 °C during 20 min. Finally, cans were sprayed with cold water to reach 30 °C. This applesauce was named non-covalent bond purée: Pnc.

For preparation of the purée with covalent bonds between procyanidins and cell walls, the above protocol was repeated until the thermal treatment at 95 °C for 1 min. Then, 200 mL of a citric acid solution at 1 kg/L was added in order to decrease the pH from 3.3 to 2.9. Purée was then crushed using a colloidal blender (Fryma, Rheinfelden, Switzerland) and then transferred back in the Stephan before deaeration under vacuum (−0.85 b) with stirring for 3 min. Apple purée was hot conditioned in 80 g-cans and pasteurized at 110 °C during 30 min. Finally, cans were sprayed with cold water to reach 30 °C. This apple purée was named covalent bond purée: Pcov.

#### 2.2.3. Extensive Rinsing of Apple Purées

In order to remove highly fermentable sugars, the two apple purées were extensively rinsed with ethanol. Each apple purée (1 kg) was suspended in ethanol: water (1 L, 80:20, *v*/*v*), agitated for 15 min, and filtrated on a G3 sintered glass filter. The apple purée was rinsed until an absence of sugars was detected in the eluate by the phenol-sulphuric test [16]. For each apple purée, eluates were pooled, concentrated on a rotary evaporator, and freeze-dried and the residue of ethanolic extraction was dried in an oven at 40 °C. After freeze-drying, phenolic compounds were purified according to the method described below. Each phenolic fraction and residue were then combined in water in order to reconstitute apple matrices without sugars, freeze-dried, and the matrices were named Mnc and Mcov respectively from the Pnc and Pcov purées.

#### 2.2.4. Extraction and Purification of the Phenolic Fraction

Phenolic compounds were extracted from the freeze-dried apple powder (150 g, mixture of 75 g of Bedan and 75 g of Reinette de Flandre) by stirring with 1 L of a water: acetone mixture (40:60 *v*:*v*) (three times) during 15 min. Extracts were pooled and concentrated on a rotary evaporator prior to freeze-drying. The extraction was repeated 7 times.

The freeze-dried extracts were dissolved in acidified water (acetic acid 25 mL/L) and filtered on a 3 µm filter (Cellulose, Merck Millipore, Darmstadt, Germany). They were injected on a 20 × 5 cm column of LiChrospher 100 RP-18 (12 µm) (Merck, Darmstadt, Germany). The column was first washed by acidified water until absence of sugars detected in the eluate by the phenol-sulphuric test [16], then a gradient of acetonitrile was applied. The eluate was monitored by absorbance at 280 nm for the presence of polyphenols. Peaks were collected and concentrated on a rotary evaporator prior to freeze-drying. The phenolic fraction was stored under vacuum at −80 °C before use.

#### 2.2.5. Preparation of Cell Wall Material

Alcohol-insoluble solids (AIS) were prepared according to Renard (2005) from freeze-dried apple powder from the two cultivars (Bedan and Reinette de Flandre) in order to obtained purified cell walls. 

#### 2.2.6. Assembly of the Mno Matrix

Based on the composition in polyphenols and cell walls of Mnc and Mcov matrices, a mix of purified cell walls and phenolic fraction was made to generate a matrix with no linkages between the two entities hereafter called Mno.

### 2.3. In Vitro Batch Fermentation and Sample Collection

Fecal donors, two males and two females, were in good health and aged between 30 and 50. They had not received any medication (antibiotics or other drugs) for at least 3 months prior to stool collection, had not knowingly consumed pre- or probiotic supplements prior to experiment, and had no history of digestive or extra-digestive diseases. The four healthy donors were informed of the study aims and procedures and provided their written consent for their fecal matter to be used for the experiments. Each subject provided stools 3 times with one week interval which made in total 12 fecal samples i.e., 4 subjects × 3 biological replicates. This was a non-interventional study with no additions to usual clinical care. According to French Health Public Law (CSP Art L 1121-1.1), such a protocol does not require approval of an ethics committee.

In vitro batch fermentations were conducted with human fecal microbiota (*n* = 12) for 48 h as follows. Fresh human fecal samples were collected in an anaerobic jar and processed within 1 h. The following steps were handled in an anaerobic chamber under 100% carbon dioxide atmosphere (Jacomex, Lyon, France). For each stool, three 250 mL-flasks equipped with screw caps GL 45 with 2 olives with EPDM gasket (Dutscher, Brumath, France) were used to incubate the fecal microbiota with the apple matrices (Mno, Mcov, Mnc) and to withdraw gas and liquid samples during fermentation. One additional flask was prepared to incubate the fecal microbiota alone in order to monitor the production of microbial metabolites that are not originating from the fermentation of the apple matrices. For each flask, ten grams of freshly collected feces were homogenized with 100 mL sterile reduced 100 mM saline phosphate buffer [17] to make 10% *w*/*v* fecal suspension. Under these conditions, the pH of the liquid phase was maintained between 6 and 7 throughout the experiment. The powders of apple matrices (Mno, Mcov or Mnc) (1.5 to 1.8 g) were added to the suspension to achieve concentrations equivalent to 1% *w*/*v* cell walls and 0.08% *w*/*v* total polyphenols. The flasks were sealed hermetically, taken out of the anaerobic chamber and placed at 37 °C into a rotary incubator. Batch cultures were run under a light agitation of 100 rpm for a period of 48 h, during which 4 mL aliquots of fecal suspension were withdrawn at 9 time points (0, 2, 4, 6, 8, 10, 24, 30 and 48 h) and stored at −20 °C for biochemical analyses. At two time points (0 and 48 h), pellets obtained by centrifugation (10,000× *g*, 4 °C, 15 min) of the fecal suspension were stored at −80 °C for microbiota analyses (Figure S1). The supernatants at 48 h were also stored at −20 °C for inflammation analyses. 

### 2.4. Biochemical Analyses

#### 2.4.1. Cell Wall Analysis in Apple Matrix

Neutral sugars, uronic acids and methanol were analyzed as described [18]. The degree of methylation (DM) was calculated as the molar ratio of methanol to uronic acid.

#### 2.4.2. Polyphenol Analysis in Apple Matrices

Polyphenols were measured by HPLC-DAD after or not thioacidolysis using a method described by Guyot et al. [19]. Their characterization and quantification were performed using an Ultra Fast Liquid Chromatography Prominence system (Shimadzu, Kyoto, Japan) controlled by the LabSolutions software (Version 5.57, Shimadzu, Kyoto, Japan). Separation conditions, identification and quantification were performed as decribed by Le Bourvellec et al. [20]. 

#### 2.4.3. Polyphenol Analysis in Fecal Slurries

Thioacidolysis was performed on the freeze-dried raw fermentation samples according to the procedure established on apple matrix samples, except that the amount of added concentrated HCl was increased to take into account the buffering capacity of the growth medium. Precisely weighted amounts (15–25 mg) of samples were placed in 1.5 mL Eppendorf vials with 150 µL of dried methanol acidified by 3N HCl and 250 µL of toluene-α-thiol solution (5%, *v*/*v* in dried methanol) were added. The reaction was carried out at 40 °C for 30 min with agitation on a vortex every 10 min. Then, the vials were cooled in an ice bath for at least 5 min. After filtration (PTFE, 0.45 µm), the medium was directly injected (20 µL) into the HPLC-DAD system.

HPLC-DAD analysis without thiolysis was also performed. Precisely weighted amounts (15–25 mg) of samples were placed in 1.5 mL Eppendorf vial with 400 µL of dried methanol acidified by acetic acid (1% *v*/*v*). The reaction was carried out in an ultrasonic bath during 15 min. After filtration (PTFE, 0.45 µm), the medium was directly injected (20 µL) into the HPLC system. The HPLC system used was as described above.

#### 2.4.4. Analysis of Microbial Metabolites

The method used to extract phenolic acids and aromatic compounds in fecal slurries was adapted from Stalmach et al. [21]. Small volumes of fecal slurries (from 400 to 900 µL) were added to a glass vial with 65 µL of 1M HCl and 100 µL of 2,4,5-trimethoxycinnamic acid (0.45 mg mL^−1^) used as internal standard. Samples were extracted three times by adding 1.5 mL of ethyl acetate, followed each time by 30 s of vortexing and centrifugation at 4000× *g* for 10 min at 4 °C. Supernatants were pooled, placed in another glass vial and dried under a flow of nitrogen. Dichloromethane (200 µL) was added to each vial and further dried under nitrogen. Samples were then derivatized by addition of 50 µL of BSTFA + 10% TMCS, and each vial was flushed with nitrogen prior to capping. The extracts were incubated at 70 °C for 2.5 h, with vortexing every 30 min to facilitate silylation. Finally, 350 µL of anhydrous hexane were added into each vial, vortexed and left to cool to room temperature prior to 1 µL being analyzed by GC-MS. The trimethylsilyl derivatives were analyzed using a QP 2010 single quadrupole GC-MS equipped with an AOC 5000 combi-pal autosampler (Shimadzu, Tokyo, Japan). Samples were injected in the split mode with a 15:1 ratio. The injector temperature was maintained at 250 °C. The mass spectrometer was used in the positive ionization mode with the ion source and transfer line set at 200 °C and 300 °C, respectively. Separations were carried out on a fused silica capillary column ZB-5MS (30 m × 0.25 mm i.d.) coated with cross-linked 5% phenylmethylsiloxane (film thickness 0.25 µm) (Phenomenex, Macclesfield, Cheshire, United Kingdom). Helium was the carrier gas with a flow rate of 1 mL min^−1^. The column temperature was initially set at 50 °C and raised to 160 °C at 20 °C min^−1^, 200 °C at 1.5 °C min^−1^ and 250 °C at 10 °C min^−1^ to a final temperature of 280 °C at 20 °C min^−1^, held for 5 min. Data acquisition was performed in full scan mode (*m*/*z* 50-600) with ionization energy of 70 eV, and analysis was carried out using GCMS Solution software version 2.72 (Shimadzu, Tokyo, Japan). Phenolic acids were identified according to the mass spectra and retention times obtained from authentic standards analyzed under identical conditions. When standards were not commercially available, identification was achieved through the integrated NIST mass spectral library 2008 (Scientific Instruments Services INC., Ringoes, NJ, USA) with a confidence of 90% or above. Calibration curves of the ratio between the peak area of the standard compound of interest and the peak area of the internal standard were computed, with different known concentrations (r^2^ > 0.95). Values for phenolic acids in the fecal slurries were expressed in µM as mean values. 

Short chain fatty acid (SCFA) analysis was performed on fermentation supernatants by gas chromatography as previously described [22].

In order to determine the degradation of procyanidins and production of microbial metabolites only coming from the fermentation of the apple matrices, the concentration of procyanidins and of each metabolite obtained during in vitro incubation of the apple matrices with the fecal microbiota were subtracted with the concentrations obtained by incubating the corresponding fecal microbiota alone i.e., no matrix added. Rate constants of procyanidin degradation and metabolite production during in vitro microbial fermentations were calculated according to a fractional conversion model associated with a first order reaction:

(1) C(t)=C∞+((C0−C∞)exp(−K∗t))

*C*0 is the measured initial concentration; *C∞* is the concentration at infinite time; *C*(*t*) is the measured concentration at time *t*; *K* is indicative of constant rate during in vitro fermentation (hour^−1^), and *t* the fermentation time (hour).

### 2.5. Microbiota Analyses

#### 2.5.1. DNA Extraction

DNA was extracted from fecal pellets at 0 and 48 h time points using the QIAamp DNA Stool Mini Kit (Qiagen, Courtaboeuf, France). DNA purity was checked using a Nanodrop-1000 Spectrophotometer (Thermo Fisher Scientific, Lyon, France). Its integrity was confirmed by electrophoresis on 0.7% agarose gels. It was quantified by using the Qubit dsDNA BR Assay Kit (Thermo Fisher Scientific, Lyon, France). 

#### 2.5.2. Sequencing Strategy and Data Analysis

Approximately 300 ng of DNA from each sample 0 and 48 h (*n* = 24) were sent to Roy J. Carver Biotechnology Center (Urbana, IL61801, USA) for fluidigm amplification targeting a ~570-bp fragment of the V3–V5 region of the 16S rRNA gene using the specific bacterial primer set V3_F357 (5′-CCTACGGGAGGCAGCAG-3′) and V5_R926 (5′-CCGTCAATTCMTTTRAGT-3′) with overhang Illumina adapters. The amplicon libraries were sequenced (along with other independent libraries) on one lane of the Illumina HiSeq 2500 platform (250 nt, paired-end) and generated approximately 185,000 ± 47,000 raw reads per sample, except for one sample having less than 1000 reads that was discarded. All sequence data are available in the NCBI Sequence Read Archive under the BioProject accession number PRJNA506944.

Sequencing data were analyzed using the IM Tornado, a workflow known as Illinois Mayo Taxon Organization from RNA Dataset Operations (http://sourceforge.net/projects/imtornado) (v2.0.2.3) and designed for R1 and R2 sequences that do not overlap [23]. The pipeline included quality filtering, merging of reads, length trimming, concatenation, read clustering after removal of short and chimeric reads, and picking of representative Operational Taxonomic Units (OTUs). The resulting OTU table was filtered to retain OTUs present in at least 50% of all samples and harboring at least 2 counts in a given sample. Taxonomy assignment was based on SILVA v123 database [24]. Taxonomic ranks assigned with a bootstrap value below 0.95 were defined as “unclassified”. 

#### 2.5.3. Quantitative Real-Time PCR (qPCR)

Quantitative PCR (qPCR) targeting the 16S rRNA gene of 4 microbial groups was performed using the QuantiFast SYBR Green PCR Kit (Qiagen, Courtaboeuf, France) and a Rotor-Gene Q device (Qiagen, Courtaboeuf, France). We used qPCR conditions and primers that were previously described for *Bacteroides* spp., *Roseburia* spp./*Eubacterium rectale*, *Faecalibacterium prausnitzii* [25] and *Bifidobacterium* spp. [26]. Standard curves were prepared according to Mosoni et al. [27] using the 16S rDNA amplicon of *Bacteroides uniformis* DSM 6597, *Roseburia intestinalis* DSM 14610, *Faecalibacterium prausnitzii* DSM 17667 and *Bifidobacterium adolescentis* DSM 20083. All qPCRs were performed in duplicate from one DNA extract per sample. 

### 2.6. Inflammation Assays

The HT-29 cell line was maintained in DMEM media supplemented with 10% fetal bovine serum, 1% glutamine and 0.1% streptomycin/penicillin at 37 °C and 10% CO_2_. Cells were washed in DPBS and trypsinised. A suspension was prepared at the concentration of 0.1 × 10^6^ cells/mL in DMEM (as described above), placing 500 µL per well in a 24 wells plate. The plate was incubated at 37 °C and 10% CO_2_ for 72 h. The medium was then changed each 24 h during 4 days. One day before the co-incubation, the medium was prepared with a reduction of fetal bovine serum concentration to 5%. In the day of the co-incubation, the medium was removed and replaced by 450 µL of DMEM medium supplemented with 5% fetal bovine serum, 1% glutamine, 0.1% streptomycin/penicillin and TNFα (5 ng/mL). 50 µL of the samples (fecal water) (final concentration 1/10) was added for each well (the samples were pre-treated with TRIS 1 M pH 7 for pH correction). We prepared two positive control wells by adding 50 µL of PBS and two negative control wells with media without TNFα and 50 µL of PBS. The plate was incubated at 37 °C and 10% CO_2_ for 6 h. The supernatant was then collected from the wells and centrifuged at 13,000× *g* for 10 min at 4 °C. After the centrifugation, the supernatant was frozen at −80 °C until the dosage of IL-8 by ELISA.

### 2.7. Statistical Analyses

Metabolite concentrations are presented as mean values, and the reproducibility of the results is expressed as pooled standard deviation. Pooled standard deviations were calculated for each series of replicates using the sum of individual variances weighted by the sum of the individual degrees of freedom [28]. Analysis of variance (ANOVA) and analysis of variance for paired data were performed using the Excelstat package of Microsoft Excel.

Microbial α-diversity metrics, such as observed OTU diversity, Chao estimates of total diversity, and Shannon and inverse Simpson diversity indices were calculated in R (https://www.R-project.org/) with the Phyloseq R package version 1.22.3 (R Foundation for Statistical Computing, Vienna, Austria) [29]. The Kruskal-Wallis rank sum test was used to assess whether or not there were differences in diversity between experimental conditions (0 or 48 h incubation with the apple matrices) and between subjects. To estimate β-diversity, the permutational multivariate analysis of variance using distance matrices (permanova) [30] from vegan R package (https://www.R-project.org/) [31] was used to determinate R-squared of OTU distance matrices and homogeneity of group variances [32]. Community structures were compared between subjects and between experimental conditions using the Bray-Curtis dissimilarity index [33] and Non-Metric MultiDimentional Scaling of distance matrix (NMDS) [31]. The differential abundance of genera and OTUs between experimental conditions was assessed using differential expression analysis based on the Negative Binomial distribution with Wald test from the DESeq2 R extension within the Phyloseq R package version 1.22.3 [29,34,35]. The effect of experimental conditions on the abundance of qPCR-targeted bacterial groups and on the inflammation response (IL-8) was estimated with a linear mixed-effect model using maximum likelihood and the lmer function from the R package lme4 [36]. Subjects were set as random effect (not controlled), and apple matrices were set as fixed effect (controlled). 

## 3. Results

### 3.1. Characterization of the Apple Matrices

#### 3.1.1. Phenolic Composition

The phenolic composition of the three apple matrices is shown in Table 1. Five phenolic groups, i.e., flavan-3-ols, dihydrochalcones, hydroxycinnamic acids, flavonols and anthocyanins, with a total of 16 identified individual compounds were quantified. Procyanidins were the predominant class accounting for 51 to 64% of total phenolic compounds in apple matrices. The average degrees of polymerisation (DPn) of procyanidins were 6. The flavan-3-ol monomers were detected as (−)-epicatechin and (+)-catechin, the former being preponderant (Table 1). Hydroxycinnamic acids contributed for 18% to 24% to the total phenolic compounds. The main hydroxycinnamic acid was 5-caffeoylquinic acid. Dihydrochalcones were a minor group which represented 4 to 6% of the total phenolic compounds. Phloridzin and phloretin-2-xyloglucoside were the major constituents of this class with phloridzin being the most abundant. Flavonols, accounted for ca. 1% of the total phenolic compounds. Six quercetin glycosides were found and quantified in quercetin equivalent: quercetin 3-galactoside > quercetin 3-arabinopyranoside > quercetin 3-rhamnoside > quercetin 3-glucoside > quercetin 3-xyloside > quercetin 3-rutinoside. (Epi)catechin−cyanidin and cyanidin compounds were only detected in Mcov matrix.

#### 3.1.2. Cell Wall Composition

Table 1 summarizes the cell wall composition of the three matrices. The two main sugars were glucose and galacturonic acid. Degrees of methylation were high, from 78% (Mcov) to 81% (Mno). Besides glucose and galacturonic acid, the neutral sugars arabinose, galactose and xylose dominated. Rhamnose, mannose and fucose were minor compounds.

### 3.2. Metabolism of the Apple Matrices during In Vitro Fermentation with Fecal Microbiota 

#### 3.2.1. SCFA Production

In order to determine the effect of the apple matrices and, more specifically, of the bond characteristics between procyanidins and cell walls on the metabolism of the latter by the fecal microbiota, the production of Short-Chain Fatty Acids (SCFAs) was kinetically monitored from 0 to 48 h.

**Figure 1:**
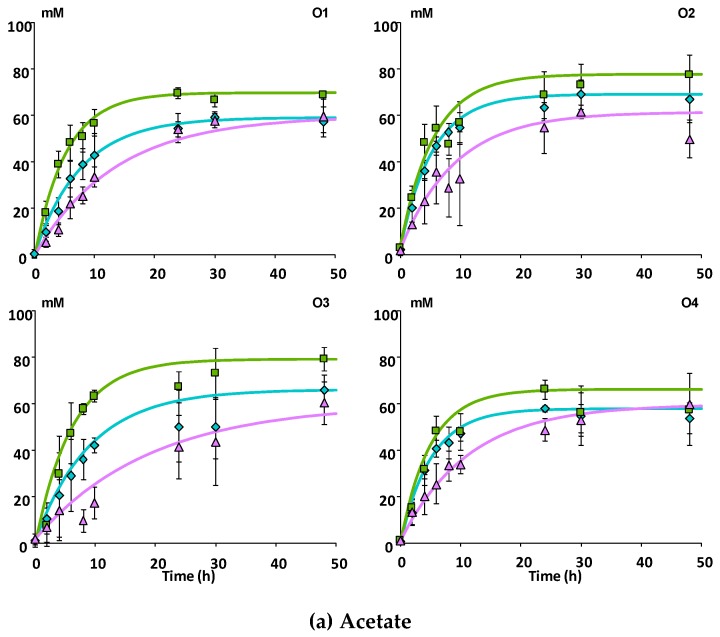

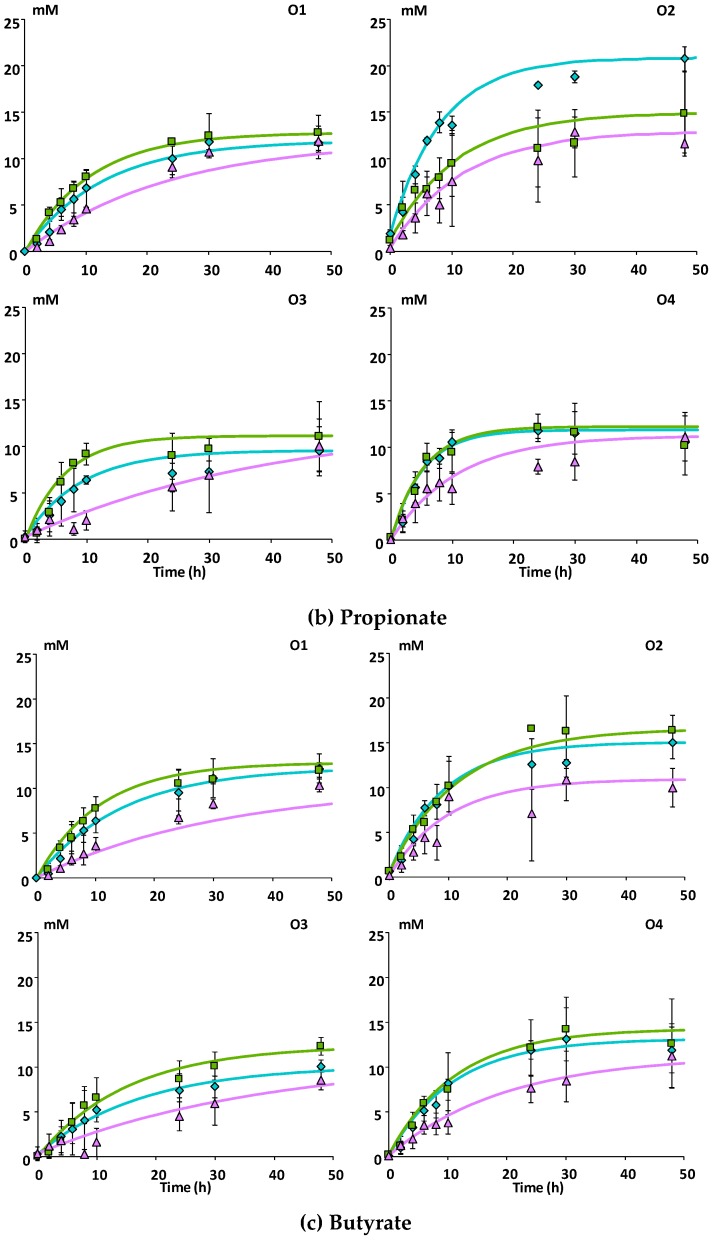
Kinetics of production of short chain fatty acids during in vitro fermentation of the apple matrices with fecal microbiota (*n* = 3) from four healthy subjects. Experimental and modelled data for acetate (**a**) propionate (**b**) and butyrate production (**c**). Each point represents the mean concentrations of SCFAs expressed in mM. Bars represent the standard error of the mean (*n* = 3). O1 to O4: subject 1 to 4. 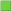 Mcov matrix, 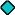 Mnc matrix, 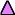 Mno matrix.

The production of the three main SCFAs i.e., acetate, propionate and butyrate is shown in Figure 1 for each matrix and each subject. Acetate was the most produced SCFA (58 ± 8 mM at 48 h) representing on average 68% of total SCFA, while propionate and butyrate were present in lower and similar concentrations (11 ± 2 mM and 10 ± 2 mM at 48 h, respectively), each representing 13–14% of total SCFA. The three SCFAs reached their maximal concentration between 24 and 48 h of incubation with Mnc and Mcov while the plateau was not reached at 48 h with Mno (Figure 1a–c). These observations correlate with the rates of SCFA production shown in Table 2. During the time course of the in vitro fermentation, the rates of acetate, propionate and butyrate production were halved (*p* ≤ 0.001) when there was no interaction between procyanidins and cell walls (Mno) compared with the two other matrices (Table 2). Furthermore, rates of acetate and propionate formation were significantly (*p* ≤ 0.05) different among subjects unlike butyrate (Table 2). Subject O3 showed the lowest acetate production rate whereas subject O1 showed the lowest propionate production rate. After 48 h, the extent of acetate and butyrate were significantly (*p* ≤ 0.01) lower with the Mno matrix than with the two other matrices (Table 3).

#### 3.2.2. Catabolism of Native Phenolic Compounds and Production of Microbial Phenolic Metabolites 

In order to follow the in vitro catabolism of phenolic compounds during the time course of incubation of the apple matrices with fecal microbiota, both native phenolic compounds and microbial phenolic metabolites were analyzed.

**Figure 2:**
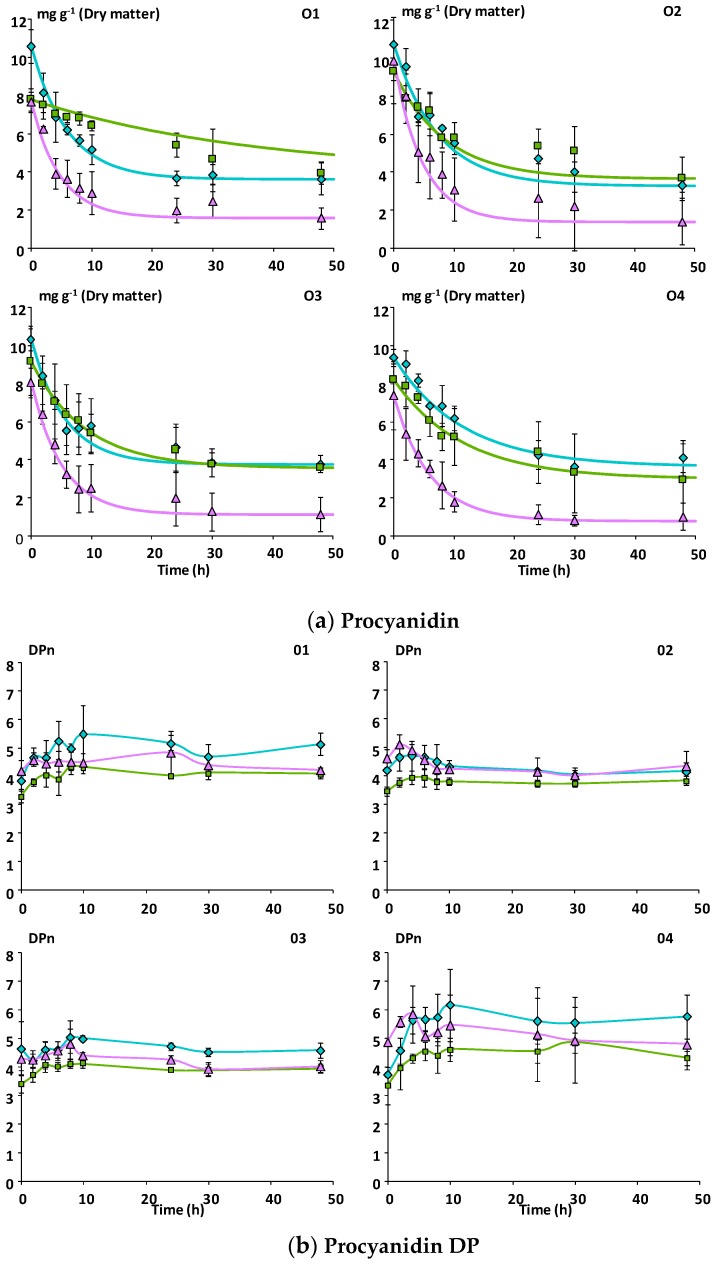
Kinetics of degradation of procyanidins during in vitro fermentation of the apple matrices with fecal microbiota (*n* = 3) from four healthy subjects. (**a**) Experimental and modelled data for procyanidin degradation. (**b**) Average degree of polymerization of procyanidins. Error bars represent the standard error of the mean (*n* = 3). O1 to O4: subject 1 to 4. 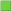 Mcov matrix, 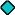 Mnc matrix, 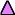 Mno matrix.

Whatever the matrix, flavan-3-ol monomers, hydroxycinnamic acids, and flavonols were no longer detected within the first four hours of incubation, indicating that they were highly susceptible to microbial degradation (data not shown). In contrast, a progressive decline in the concentration of oligomeric procyanidins was observed, with degradation profiles being similar for the four subjects (Figure 2a). On average 84% (± 16) of procyanidins were degraded from Mno after 48 h incubation whereas only 64% (± 7) and 58% (± 9) were degraded from Mnc and Mcov, respectively. The differences were significant (*p* ≤ 0.001) only between Mno and the two other matrices (Table 3). Accordingly, the rates of procyanidin degradation were significantly (*p* ≤ 0.001) higher with Mno and no differences between subjects were observed (Table 2). Furthermore, whatever the matrix and the subject, the degree of polymerization of residual procyanidins increased slightly until 10 h incubation, and then decreased slightly to stabilize at the end of fermentation (Figure 2b).

**Figure 3:**
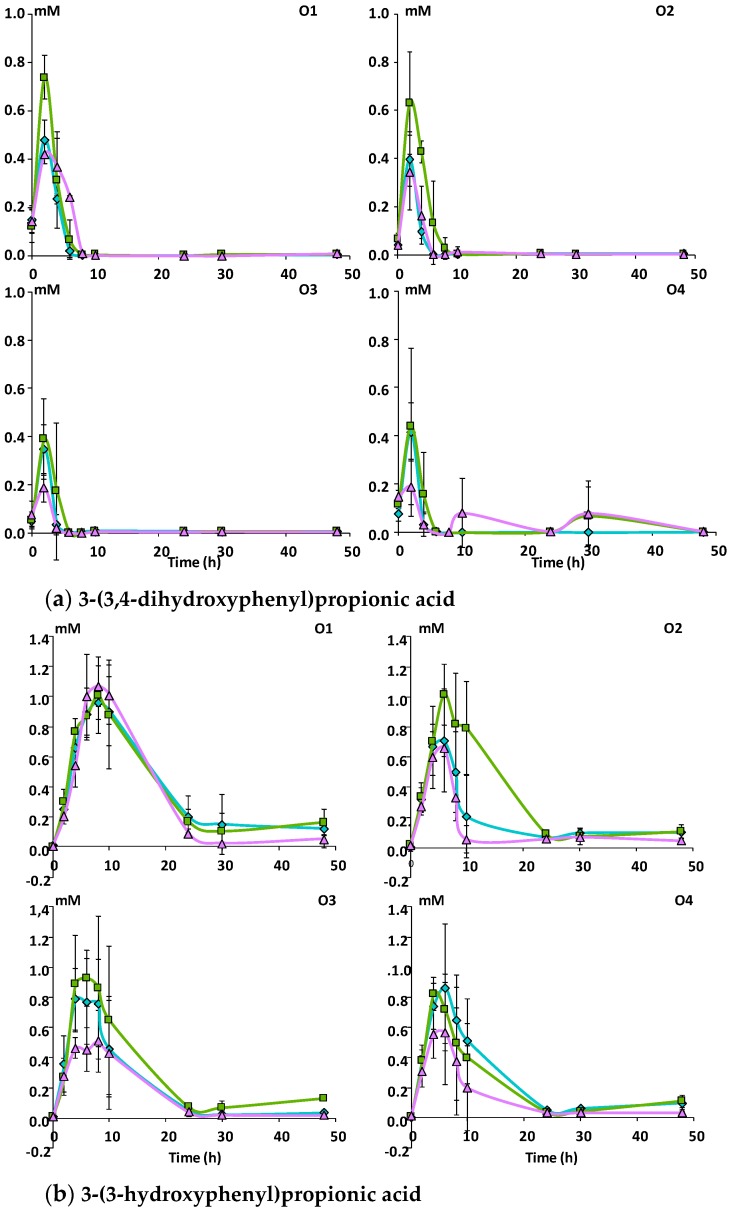

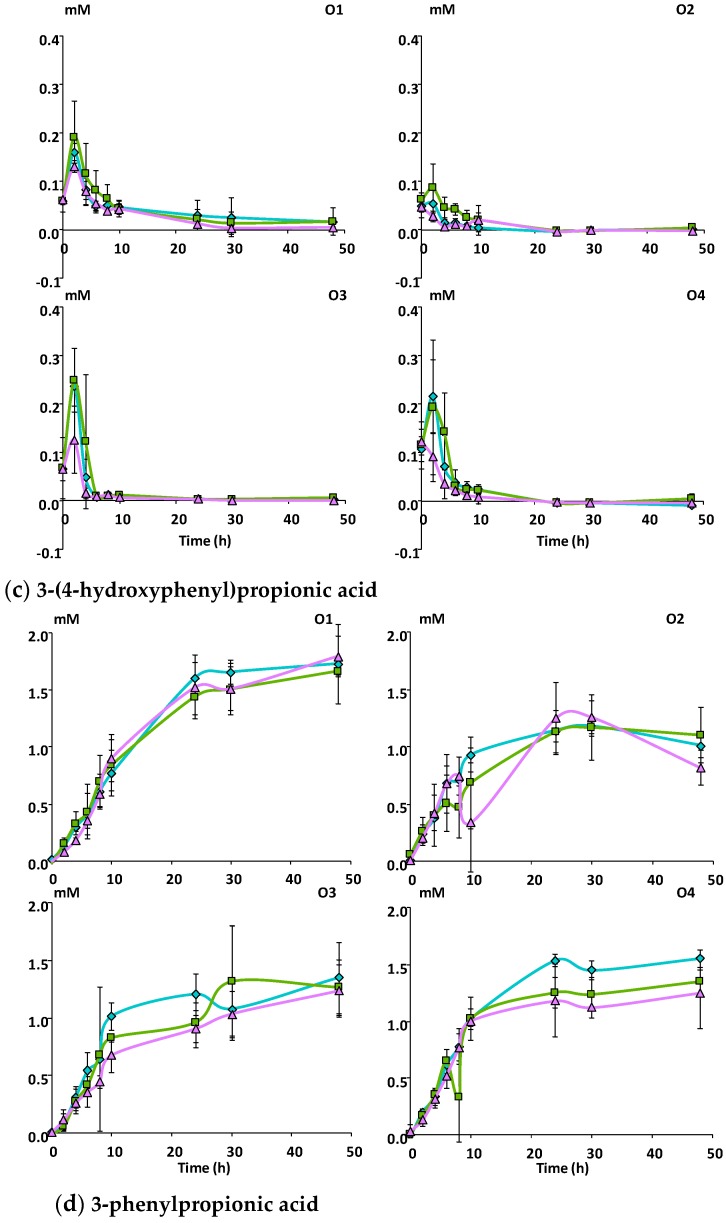
Kinetics of production of phenylpropionic acid derivatives during in vitro fermentation of the apple matrices with fecal microbiota (*n* = 3) from four healthy subjects. Mean concentrations in mM of (**a**) 3-(3,4-dihydroxyphenyl)propionic acid, (**b**) 3-(3-hydroxyphenyl)propionic acid, (**c**) 3-(4-hydroxyphenyl)propionic acid, (**d**) 3-phenylpropionic acid. Error bars represent the standard error of the mean (*n* = 3). O1 to O4: subject 1 to 4. 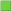 Mcov matrix, 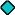 Mnc matrix, 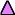 Mno matrix.

**Figure 4:**
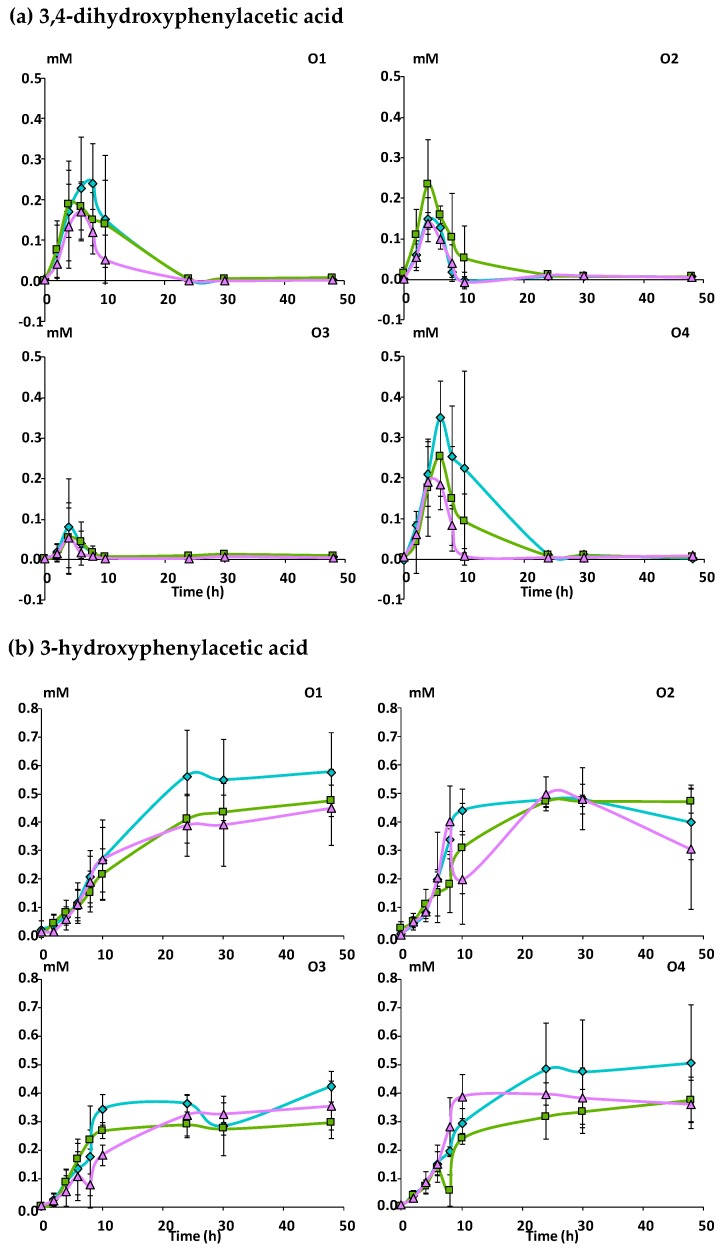
Kinetics of production of phenylacetic acid derivatives during in vitro fermentation of the apple matrices with fecal microbiota (*n* = 3) from four healthy subjects. Mean concentrations in mM of (**a**) 3,4-dihydroxyphenylacetic acid and (**b**) 3-hydroxyphenylacetic acid. Error bars represent the standard error of the mean (*n* = 3). O1 to O4: subject 1 to 4. 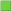 Mcov matrix, 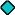 Mnc matrix, 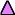 Mno matrix.

**Figure 5:**
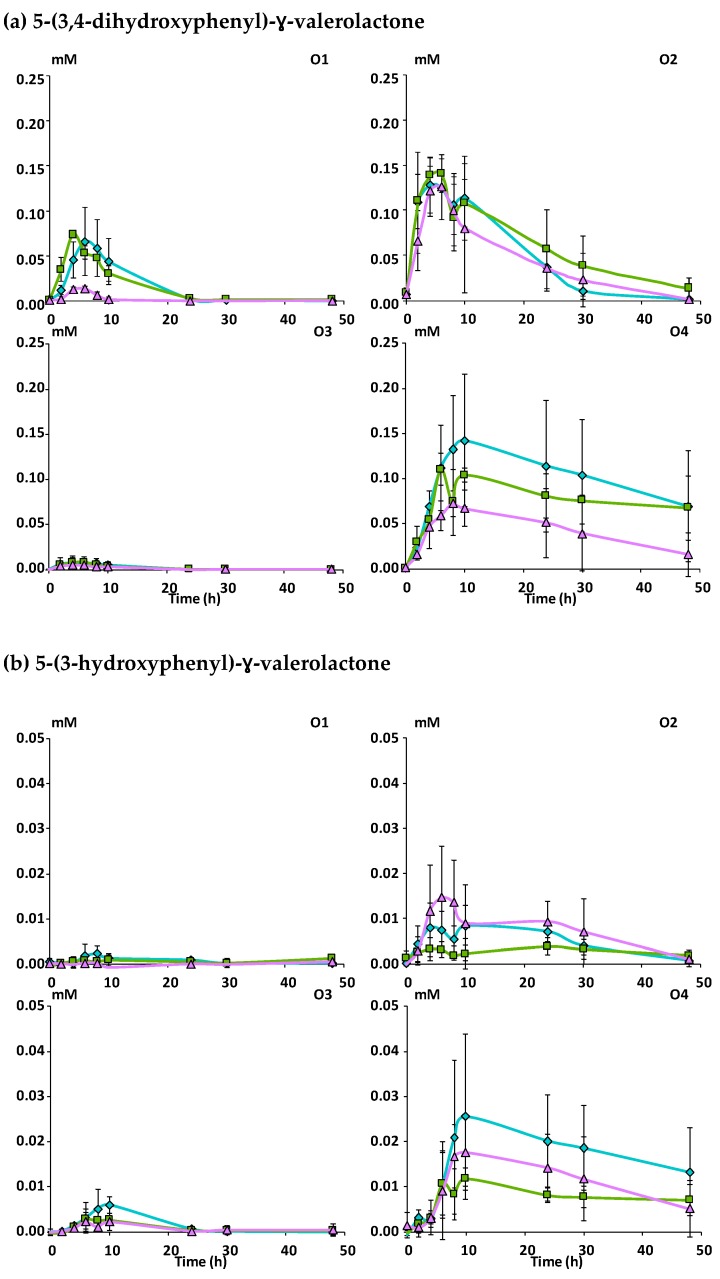

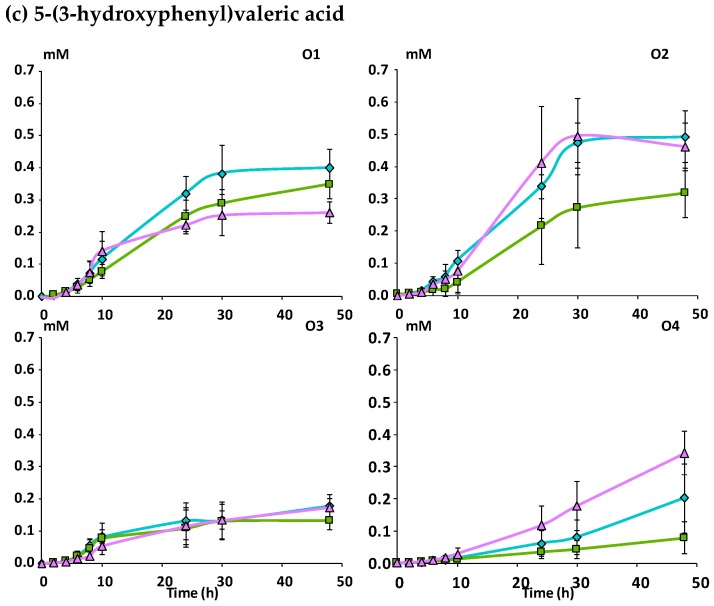
Kinetics of production of phenylvalerolactone and phenylvaleric acid derivatives during in vitro fermentation of the apple matrices with fecal microbiota (*n* = 3) from four healthy subjects. Mean concentrations in mM of (**a**) 5-(3,4-dihydroxyphenyl)-ɣ-valerolactone, (**b**) 5-(3-hydroxyphenyl)-ɣ-valerolactone, (**c**) 5-(3-hydroxyphenyl)valeric acid. Error bars represent the standard error of the mean (*n* = 3). O1 to O4: subject 1 to 4. 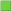 Mcov matrix, 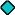 Mnc matrix, 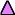 Mno matrix.

A total of 16 phenolic metabolites were identified in the fecal slurries during the incubation of the apple matrices with fecal microbiota (Table S1). 3-phenylpropionic acid and its hydroxylated derivatives were the most abundant metabolites (Figure 3) followed by phenylacetic acid and its 3-hydroxylated derivative (Figure 4) and by the 5-phenyl-ɣ-valerolactones and 5-phenylvaleric acid derivatives (Figure 5). Benzoic acid, phenyllactic acid and their hydroxylated derivatives were also produced but in lower amounts (data not shown).

The phenylpropionic acid metabolites had different kinetic profiles during the time course of fermentation. Whatever the matrix and the subject, 3-(3,4-dihydroxyphenyl)propionic acid increased and reached a peak after 2 h of fermentation and then decreased rapidly (Figure 3a). 3-(3-hydroxyphenyl)propionic acid increased for 6 hours and then slowly decreased (Figure 3b). 3-(4-hydroxyphenyl)propionic acid appeared and disappeared rapidly, in a similar manner as 3-(3,4-dihydroxyphenyl)propionic acid (Figure 3c). On the other hand, phenylpropionic acid increased progressively during the first 20 h of incubation and accumulated in the medium (Figure 3d). A higher production (*p* ≤ 0.05) of 3-(3,4-dihydroxyphenyl)propionic, 3-(3-hydroxyphenyl)propionic and 3-(4-hydroxyphenyl)propionic acids was observed with Mcov, followed by Mnc and then Mno, but there was no significant differences in phenylpropionic acid concentration among matrices at 48 h (Table 3). Significant differences (*p* ≤ 0.05) among subjects were also observed in the maximal concentrations of this series of metabolites (Table 3). Subject O1 presented the highest production of 3-(3,4-dihydroxyphenyl)propionic, 3-(3-hydroxyphenyl)propionic and phenylpropionic acids whereas subject O3 showed the highest production of 3-(4-hydroxyphenyl)propionic acid (Figure 3). 

Within the phenylacetic acid derivatives, 3,4-dihydroxyphenylacetic acid was an intermediate product whose concentration reached a peak between 4 and 6 h of fermentation and then decreased rapidly with Mno while more gradually with the two other matrices (Figure 4a). 3-hydroxyphenylacetic acid, increased and reached a plateau after 10 to 24 h of fermentation with no further degradation (Figure 4b). Significant differences among the three matrices (Mnc > Mcov > Mno; *p* ≤ 0.05) and the four subjects (O4 > O1 > O2 > O3; *p* ≤ 0.001) were observed for the intermediate metabolite i.e., 3,4-dihydroxyphenylacetic acid, but not for the end-product i.e., 3-hydroxyphenylacetic acid. 

Among the remaining identified phenolic metabolites, 5-(3,4-dihydroxyphenyl)-ɣ-valerolactone increased and reached a maximum level after 6 hours of fermentation for subjects O1, O2 and O3, and 10 h for O4, and then decreased slowly (Figure 5a). Its maximal concentration showed a large inter-individual variation (*p* ≤ 0.001) (Table 3), subjects O2 and O4 being the largest producers followed by O1 and O3. Dehydroxylation of 5-(3,4-dihydroxyphenyl)-ɣ-valerolactone led to the formation of 5-(3-hydroxyphenyl)-ɣ-valerolactone within 6 to 10 hours of fermentation depending on the subject to finally decline slowly (Figure 5b). Like for its precursor, large inter-individual variations were observed (*p* ≤ 0.001, Table 3), subject O4 being the largest producer. Finally, 5-(3-hydroxyphenyl)valeric acid was produced at higher concentrations than the previous transitory metabolites and accumulated in the medium after 30 h of fermentation (Figure 5c). A lower production (*p* ≤ 0.01) of this end-product was observed for Mcov despite a large inter-individual variation, subject O2 being the largest producer and O3 the smallest (Table 3).

All the phenolic metabolites described above were present in the original fecal suspensions at T0 with concentrations ranging from 0.002 to 0.2mM (Figure S2). 48 h metabolization of the apple matrices induced at least a 10-fold increase in the concentrations of the end-products i.e., phenylpropionic acid, 3-hydroxyphenylacetic acid, 5-(3-hydroxyphenyl)valeric acid, while the other metabolites were in the same range of concentrations as in the original fecal suspensions (Figure S2).

### 3.3. Bacterial Community Composition upon Growth on the Apple Matrices

Fresh fecal samples from four healthy subjects (O1, O2, O3, O4) were incubated for 48 h in batch fermenters with apple matrices (Mcov, Mnc or Mno) or no matrix (Figure S1). The latter condition, carried out to calculate the net production of metabolites from the apple matrices (See Material & methods), was not considered for microbiota analyses. Indeed, with no matrix, fermentation was extremely low (data not shown), the resulting microbiota after 48 h incubation reflecting a “fasting” microbiota whose composition was not relevant to our study. 

In consequence, in order to investigate both the matrix effect and the procyanidin-cell wall linkage effect on the bacterial community composition, the analyses were performed at the initial (Control T0) and final (T48 h) time points of batch incubation of the fecal microbiota with the apple matrices (Mcov, Mnc, Mno). In total, 48 samples were analyzed by bacterial 16S metabarcoding (4 subjects × 3 feces × 4 conditions i.e., Control-T0, Mcov-T48, Mnc-T48, Mno-T48) (Figure S1). One sample from subject O2 (Mnc-T48) was discarded because less than 1000 sequences were obtained for this sample. 

**Figure 6:**
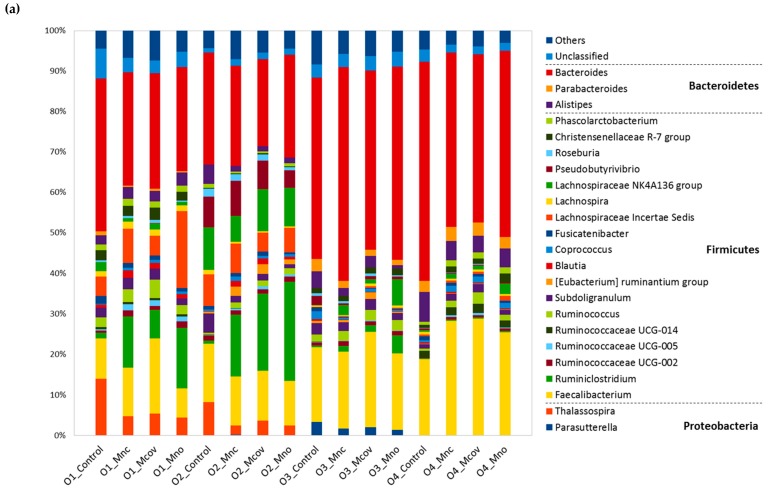

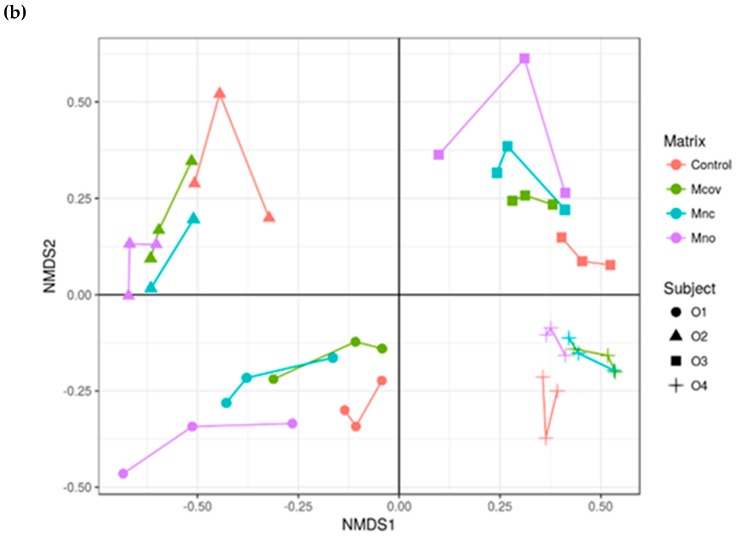
Genus-level relative bacterial composition of fecal slurries at the initial time point of fermentation (Control) and after 48 h fermentation of the three apple matrices (Mcov, Mnc, Mno). (**a**) Relative abundances of the top 23 classified genera sorted by phylum are depicted, with the genera represented by less than 5000 reads pooled into “Others”. (**b**) Genus-level bacterial community variation represented by Non-metric MultiDimenstional Scaling (Bray-Curtis dissimilarity). Samples (*n* = 47) were colored and shaped according to experimental conditions (Control + Matrices) and subject, respectively. The analysis of variance using distance matrices gave a R-squared of 0.08 in response to matrix and a R-squared of 0.66 in response to subject (*p* value < 0.0001).

On the 47 remaining samples, a total of 310 OTUs was obtained (Table S2). When looking at the initial microbiota composition at the genus level (Control T0), all subjects harbored a very high relative abundance of *Bacteroides* (28 to 54%), *Faecalibacterium* (10 to 19%) belonging to the Bacteroidetes and Firmicutes phylum, respectively (Figure 6a). Other bacterial groups or genera were also relatively abundant (0.1 to 10% depending on subjects) in the Firmicutes (*Ruminiclostridium, Ruminococcaceae*_UCG-002, *Ruminococcus*, *Subdoligranulum*, *Blautia, Coprococcus*, *Fusicatenibacter*, *Lachnospira*, *Lachnospiraceae*_NK4A136_group, *Roseburia*, *Christensenellaceae*_R-7_group) and the Bacteroidetes (*Alistipes*). Some bacterial groups or genera were either absent or at high levels (>3%) depending on subjects, like *Pseudobutyrivibrio* (Firmicutes) *Parabacteroides* (Bacteroidetes) or *Thalassospira* and *Parasuterella* (Proteobacteria). 

Regarding β-diversity, both subject and matrix impacted significantly the microbiota composition at the genus level (PERMANOVA, *p* < 0.0001). The main variation (67% of total variation) was due to inter-individual differences; apple matrices only induced 8% of total variation (Figure 6b). No “matrix” effect was observed on the absolute numbers of the four bacterial groups targeted by qPCR i.e., *Bifidobacteria*, *Roseburia-Eubacterium rectale, Faecalibacterium prausnitzii* and *Bacteroides* (Table S3). In addition, the apple matrices did not affect bacterial α-diversity, as assessed by observed OTU richness, Chao estimates of total richness, and the Shannon and Inverse Simpson diversity indices (both reflecting richness and evenness) (Kruskall Wallis test, *p*  >  0.05) (Figure S3a). α-Diversity was however different from one subject to another (Kruskall Wallis test, *p*  <  0.0005), with subject O1 harboring the greatest richness and evenness and subject O2 harboring the lowest richness (Figure S3b). 

**Figure 7:**
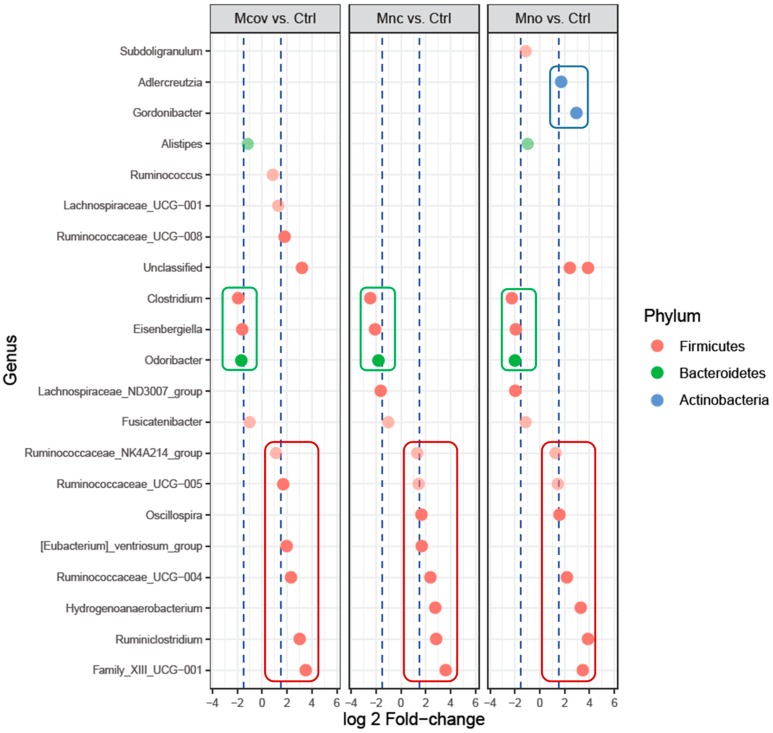
Differential genus abundance analysis between microbiota after fermentation of the apple matrices (Mno, Mnc, Mcov) and initial microbiota (control T0). Log2 Fold-Change relative abundance of 22 differentially abundant genera (adjusted *p*-value < 0.05). The x-axis and dot colors refer to the genus and phylum assignment, respectively. Genus abundances positively or negatively affected by the 3 matrices were surrounded by red and green circles, respectively, and genera specifically enriched with 1 matrix were surrounded by blue circles.

Pairwise comparisons using DESeq2 (Mnc versus Mno, Mcov versus Mno) did not underline any differentially abundant genera between Mnc and Mno or between Mcov and Mno, showing no effect of procyanidin-cell wall linkages on the microbiota composition (not shown). Nevertheless, pairwise comparison between Mcov, Mnc, Mno microbiota (T48) and initial microbiota (control T0) underlined 22 differentially abundant genera (19 Firmicutes + 1 Bacteroidetes + 2 Actinobacteria) (Figure 7). Seven genera or bacterial groups, all Firmicutes, increased with the three matrices while three decreased (defined as Log2 Fold-Change ≥ 1.5 or ≤ −1.5, respectively). *Ruminiclostridium* and Family_XIII_UCG-001 were the ones that increased the most. Interestingly, two genera (*Adlercreutzia, Gordonibacter*) increased specifically with Mno and belonged to the *Coriobacteriaceae* family (Actinobacteria phylum). 

### 3.4. Inflammatory Response

**Figure 8:**
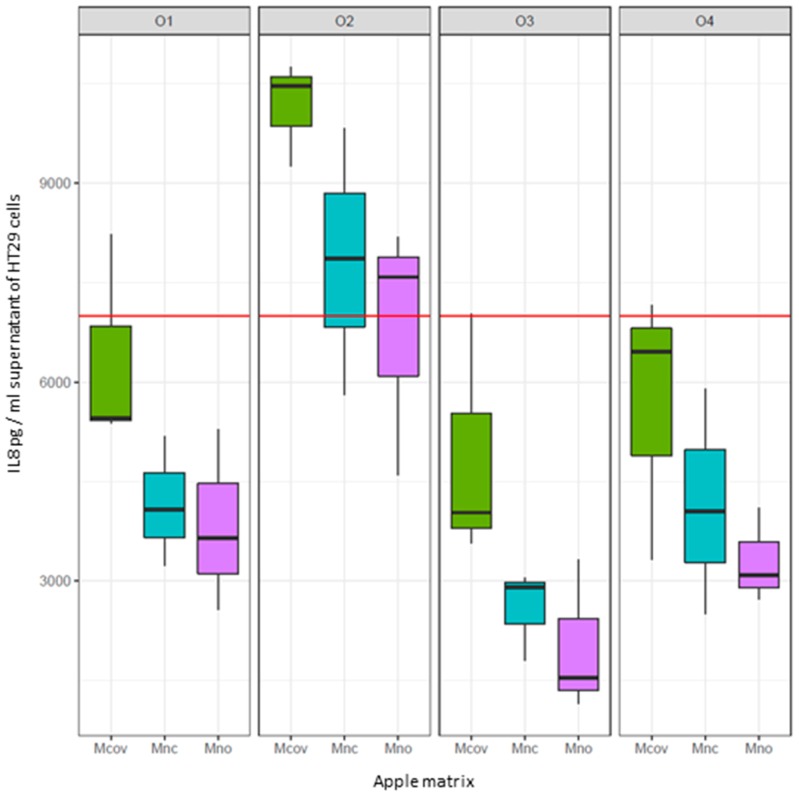
Inflammatory response of intestinal cells HT29 to fermenter supernatants according to subject and treatment (apple matrix: 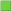 Mcov, 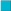 Mnc, 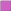 Mno). Interleukin 8 (IL8) was used as a marker of inflammation. The red line corresponds to IL8 production by TNFα-treated intestinal cells. The box plots represent IL8 production (pg/mL) by TNFα treated cells on which the supernatant of the fermenters after 48h fermentation of the apple matrices has been applied. Centre lines show the medians; box limits indicate the 25th and 75th percentiles. When IL8 levels > red line: pro-inflammatory effect of the supernatants. When IL8 levels < red line: anti-inflammatory effect of the supernatants.

HT-29 cells were stimulated by TNFα and co-incubated with or without fecal water obtained after 48 h of fermentation of the three apple matrices (Mcov, Mnc, Mno) with feces from subject O1, O2, O3 and O4. Supernatants were discarded after 6 hours of incubation and IL-8, as marker of inflammation was assayed by ELISA. Globally fecal water co-incubation resulted in a decrease of IL-8 in HT-29 supernatant except for subject O2 (Figure 8). The lowest IL-8 concentration was detected in supernatant of HT-29 cells co-incubated with Mno matrix, even for subject O2. 

## 4. Discussion

In order to compare the effect of different linkages between procyanidins and plant cell walls on the metabolism of cell wall polysaccharides and procyanidins by the human gut microbiota, we used different methods involving industrial and biochemical processes to produce apple food models that were expected from our previous studies to generate either non-covalent linkages or covalent linkages or no linkages between procyanidins and cell walls i.e., Mnc, Mcov and Mno, respectively [8,37]. Biochemical analyses were then undertaken to check that the composition of the three matrices was not dramatically different, which was a prerequisite to our experimental design. 

When looking at the phenolic composition of the three matrices, oligomeric procyanidins and 5-caffeoylquinic acid were the main contributors, in agreement with previous data reported on apple [1,2,20,38,39]. A lower procyanidin content was observed in Mcov in agreement with their chemical depolymerization in hot acidic conditions and their nucleophilic reactions with cell walls [8]. Another evidence of chemical degradation of procyanidins lies in the fact that (+)-catechin and (−)-epicatechin contents were higher in Mcov than Mnc and Mno and that tannin-anthocyanidin structures namely (epi)catechin-cyanidin and cyanidin compounds were detected [8]. Regarding cell wall composition, the three matrices were also very close. Their sugar composition reflected the macromolecular composition of apple cell walls [40,41] which contains cellulose, highly methylated pectins relatively rich in xylogalacturonans with side chains of arabinans and galactans, and hemicelluloses mainly composed of fucogalactoxyloglucan and mannans [41,42,43]. As for phenolic composition, Mcov was slightly different from the two other matrices with lower concentrations of rhamnose, arabinose and glucose. This could be due to the formation of new compounds resulting from the formation of covalent adducts between flavan-3-ols and sugars leading to an underestimation of their content. 

Overall, the three apple matrices were sufficiently equivalent in their composition to investigate the “procyanidin-cell wall linkage” effect on the metabolism of cell wall polysaccharides and procyanidins by the human gut microbiota. Consequently, we used in vitro batch fermenters and fresh fecal microbiota from four healthy subjects (feces collected in triplicate for each subject) to kinetically follow the metabolization of the three apple matrices over a period of 48 h. This allowed us to monitor (i) the production of Short Chain Fatty Acids (SCFAs) which reflected carbohydrate fermentation and (ii) the degradation of procyanidins and the production of phenolic metabolites.

Regarding carbohydrate fermentation, we first observed that the rate of production of the three main SCFAs i.e., acetate, propionate and butyrate was halved with Mno compared with Mnc and Mcov. This resulted in lower concentrations of butyrate at the end point of fermentation (48 h) with Mno. No significant differences were observed between Mcov and Mnc although Mcov tended to be slightly better fermented than Mnc. Consequently, these data show that no interactions between procyanidins and cell walls inhibit carbohydrate metabolism i.e., degradation of cell-wall polysaccharides and fermentation of the resulting sugars by the microbiota. This result is in agreement with previous studies reporting a strong inhibition of SCFA formation with free procyanidins compared with procyanidins enclosed in the apple matrix [11,13]. One explanation to this phenomenon is that free procyanidins inhibit bacterial carbohydrate-active enzymes [44] possibly because of their ability to form strong complexes with proteins [7]. Another explanation would be that cell walls in Mcov and Mnc were better degraded because of a higher cell wall porosity due to the thermal treatments to which they were submitted, contrary to the cell walls in Mno that were not heated but prepared as alcohol insoluble solid from freeze-dried apples.

Contrary to carbohydrate fermentation, procyanidins were better degraded (up to 80% degradation) when unbound to the cell walls (Mno). Indeed, both rate and extent of procyanidin degradation were reduced by the linkages between procyanidins and cell walls (Mcov and Mnc). In addition, whatever the matrix, the degree of polymerization of the residual procyanidins was quite stable during the 48 h of fermentation. Our results support previous in vitro and in vivo studies reporting that procyanidins could be degraded by the intestinal microbiota [10,45,46]. Procyanidin linked to cell walls might be less degraded because of steric hindrance between bacterial enzymes targeting procyanidins (to date unknown enzymes) and the numerous bacterial carbohydrate-active enzymes targeting cell wall polysaccharides [47]. The fact that flavan-3-ol monomers were not detected at any time point during fermentation in addition to the unchanged degree of polymerization of residual procyanidins suggest that the catabolism of procyanidins did not proceed by cleavage of the interflavan bond. These results confirm previous in vitro studies using procyanidin dimers that also strongly suggested that the main degradation route of procyanidins is not depolymerization [48,49,50,51]. Actually, several pathways of degradation of monomeric and oligomeric procyanidins by the human gut microbiota have been proposed, and might involve enzymatic reactions that catalyze C-ring opening followed by lactonization, A-ring opening, decarboxylation, dehydroxylation, and oxidation reactions, among others, leading to a large collection of phenolic metabolites [48,49,52,53]. 

**Figure 9:**
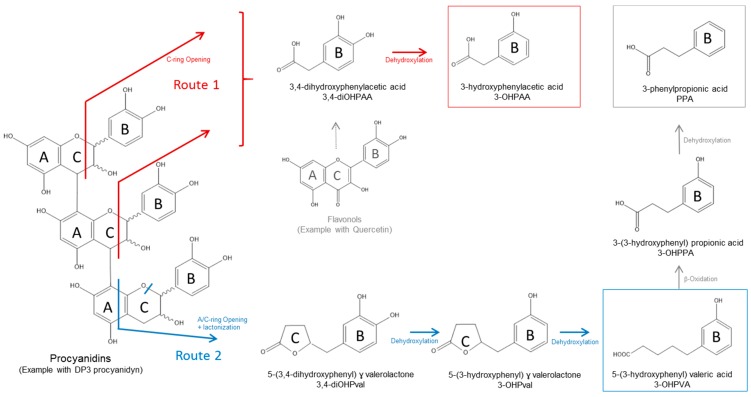
Proposed pathways for procyanidin catabolism by the human gut microbiota (adapted from Appeldoorn et al., 2009 [48] and Duenas et al., 2015 [53]). Route 1 in red and Route 2 in blue correspond to the hydrolysis pathways of the upper and lower units of procyanidins, respectively. The additional route in grey could have contributed to the formation of other detected metabolites. Metabolites in frames were the ones that reached a plateau after 48h fermentation (end-products). The other ones were transitory metabolites. Flavonols (<2% *w*/*w* of apple matrices) were added in the figure because they may contribute to the formation of hydroxyphenylacetic acid metabolites according to Del Rio et al. 2013 [54].

Within the 16 phenolic metabolites that we were able to identify in this study, most of them were transitory while three major metabolites (phenylpropionic acid, 3-hydroxyphenylacetic acid, 5-(3-hydroxyphenyl)valeric acid) and two minor metabolites (benzoic acid, phenyllactic acid, not shown) accumulated in the medium. The fecal microbiota of the four subjects were able to generate all these metabolites despite inter-individual differences both in the extent and kinetics of production. From these data, a tentative schematic pathway of apple procyanidin degradation is proposed in Figure 9. As suggested by Appeldoorn et al. [48], C-ring opening of the oligomer upper unit may lead to the production of 3,4-dihydroxyphenylacetic acid which is dehydroxylated into 3-hydroxyphenylacetic acid (end-product) (Route 1). C- and A-ring fission of the lower unit may result in the formation of 5-(3,4-dihydroxyphenyl)-ɣ-valerolactone, which is dehydroxylated to form 5-(3-hydroxyphenyl)-ɣ-valerolactone, which then leads to 5-(3-hydroxyphenyl)valeric acid (end-product) after dehydroxylation of the lactone (Route 2). Route 2 contrary to route 1 was partially inhibited by the linkages between procyanidins and cell walls since a significant lower production of 5-(3-hydroxyphenyl)valeric acid was observed with Mcov. This result confirms our assumption that procyanidins may bind covalently to cell walls via the lower units [8], hence reducing their hydrolysis by microbial enzymes. In addition, according to Appeldoorn et al. [48] and Duenas et al. [53], 5-(3-hydroxyphenyl)valeric acid may be further converted into 3-(3-hydroxyphenyl)propionic acid by α-oxidation and then into 3-phenylpropionic acid by dehydroxylation (Figure 9). The slow kinetics of disappearance of 3-(3-hydroxyphenyl) propionic acid outlined in Figure 3b compared to the other hydroxyphenylpropionic derivatives agrees with this additional route.

It is also important to note that the production of most metabolites, except 5-(3-hydroxyphenyl)valeric acid and hydroxyphenyl-ɣ-valerolactone derivatives, could not be correlated with the extent of procyanidin degradation. One explanation is that the three apple matrices also contained caffeic acid derivatives (between 18% and 24% of total polyphenols) and other minor polyphenols (<2% flavonols; 4–6% dihydrochalcones; <1.5% *p*-coumaric acid) that could be rapidly degraded (<10 h) into phenylpropionic acid derivatives as well as phenylacetic acid derivatives as shown in previous studies [10,11,13,55]. Another explanation is that procyanidin oligomers were converted into various oligomeric catabolites, as observed by Stoupi et al. [49] with procyanidin dimers, that were not detected with our analytical methods. An adaptation of the degradation pathway of apple phenolic compounds (other than procyanidins) based on the literature is proposed in Figure S4 [12,54]. Consequently, in our study, only 5-(3-hydroxyphenyl)valeric acid and its transitory precursors (hydroxyphenyl-ɣ-valerolactones) could be considered as metabolic markers of procyanidin degradation by the human gut microbiota. 

Overall, inter-individual variability was observed in the production of SCFAs and phenolic microbial metabolites but it is for the latter that the differences among subjects were the greatest, in particular for the hydroxyphenyl-ɣ-valerolactone and hydroxyphenylvaleric acid derivatives (Table 3, Figure 9). Inter-individual variability in the production profiles of these particular metabolites was already observed in vitro (fecal fermentation) and in vivo (plasma, urine) with different sources of flavan-3-ols as reviewed by Mena et al. [56]. Very recently, Mena et al. [57] have been able to discriminate three groups of individuals (putative metabotypes) from their urinary metabolome after a chronic consumption of green tea and coffee extracts rich in flavan-3-ols. Their data tend to show that there is an increasing efficiency of the microbial activity in converting tri- and dihydroxyphenyl-ɣ-valerolactones into hydroxyphenylpropionic acids. Our results obtained with apple procyanidins are in part consistent with their findings with subjects O2 and O4 being high producers of hydroxyphenyl-ɣ-valerolactones and hydroxyphenylvaleric acid, subject O1 being a medium producer and subject O3 being a low producer of these metabolites. More studies with large cohorts are needed to confirm this individual stratification and to correlate the bioaccessibility of these metabolites to their bioavailability. One must keep in mind that in vitro fermentation studies only reflect microbial metabolism and do not consider the colon dynamic where metabolites are continuously generated, absorbed and excreted.

Of course the metabolic differences between subjects reflect the high inter-individual variability of the bacterial community composition that was highlighted here through 16S sequencing analyses and that was already described in several metagenomic studies performed on large cohorts [58]. In this study, we found that the apple matrices induced very little changes in the bacterial community composition with no apparent prebiotic effect, contrary to Koutsos et al. [55] who observed an increase in Bifidobacteria with three different apple varieties. Among the changes, we identified several Firmicutes of the *Ruminococcaceae* family that increased notably with the three apple matrices. Presumably some of these family members correspond to bacteria whose growth was promoted by cell wall polysaccharides and was not inhibited by apple polyphenols. *Ruminiclostridium* genus, which was highly stimulated, is represented by very efficient polysaccharide-degrading bacteria and could belong to this category [59,60]. Although no procyanidin-cell wall linkage effect was observed when performing global analyses, pairwise comparisons allowed us to show that *Adlercreutzia* and *Gordonibacter* genera, although at very low levels in the initial fecal microbiota, increased specifically with the Mno matrix. This result is not surprising since these two genera have been shown to metabolize dietary polyphenols. *Adlercreutzia equolifaciens*, first described as an equol-forming bacterium from soy isoflavones [61], was recently shown to metabolize flavan-3-ol monomers i.e., (−)-epicatechin, (−)-catechin, and (+)-catechin by performing C-ring cleavage [62]. *Gordonibacter* genus is also strongly linked to the metabolization of polyphenols [63] with one species, *Gordonibacter faecihominis*, capable of dehydroxylating (+)-catechin derivatives [64]. Consequently, it seems reasonable to conclude that these two genera belonging to the Actinobacteria phylum participate in the metabolism of apple monomeric procyanidins. Their involvement in the bioconversion of oligomeric procyanidins, although probable since these bacteria increased specifically when procyanidins were not linked to cell walls, has yet to be demonstrated.

One last objective of this study was to investigate the anti-inflammatory effect of the metabolome resulting from 48 h in vitro metabolization of the three apple matrices by fecal microbiota, after removal of microbial cells and apple matrix residues from the liquid phase. One interesting result was that for three subjects (O1, O3, O4), it was the metabolome generated from the Mno matrix that showed the highest anti-inflammatory effect as revealed by the reduction in IL-8 response by TNFα stimulated intestinal cells. The metabolome generated from Mnc exerted a lower anti-inflammatory effect than Mno but much higher than that of Mcov. Subject O2 generated a metabolome that was pro-inflammatory but when comparing the responses between matrices, it was the Mno-metabolome that was the least pro-inflammatory. The anti-inflammatory effect of human fecal water has been already described [65]. Authors showed that fecal water from 20 human vegetarian volunteers decreased the Cox-2 and PGE2 production by TNFα stimulated HT-29 intestinal cells. Here we can see that metabolization of apple matrices can induce an anti-inflammatory activity, especially when procyanidins are not linked to cell walls. Unfortunately, we were not able to correlate this effect to any of the SCFAs and phenolic metabolites identified within the metabolome, including butyrate, which is known for its anti-inflammatory activity [66], meaning that the anti-inflammatory effect results from a combination of several metabolites, or that other unidentified molecules are involved. Further investigations are needed to identify metabolites other than butyrate that could induce an anti-inflammatory activity towards intestinal cells. This study, although performed with a limited number of subjects, shows for the first time a potential health effect of apple polyphenols through the metabolic activity of the intestinal microbiota, the effect being modulated by the linkages between procyanidins and cell walls. 

## 5. Conclusions

Using in vitro approaches, this study showed for the first time that the binding of procyanidins to apple cell-wall polysaccharides, whether covalently or non-covalently, limited procyanidin degradation by the intestinal microbiota. Unbound procyanidins were better degraded but they affected negatively carbohydrate fermentation and reduced the production of short chain fatty acids and especially butyrate, which is known for its beneficial effects on health. This study also highlighted several specific phenolic metabolites (hydroxyphenylvalerolactones, hydroxyphenylvaleric acid) that are considered as markers of intestinal microbial metabolization of flavan-3-ols. Furthermore, it underlined microbial metabolic differences among individuals towards this class of polyphenols. Finally, the best results in terms of the production of anti-inflammatory bioactive metabolites, whether from cell walls or procyanidins, were observed in the apple matrix with no bonds between the two moieties, although the matrix with non-covalent bonds was not far behind.

## Figures and Tables

**Table 1:** Composition in phenolic compounds (% *w*/*w*) and cell wall compounds (mg·g^−1^ AIS) of the studied apple matrices prepared from Bedan and Reinette de Flandre cultivars.

Apple Matrix Composition	Apple Matrices			
	Mnc	Mcov	Mno	Pooled SD ^3^
**Phenolic compounds ^1^**				
Flavan-3-ols				
CAT	4.39	5.83	4.16	0.66
EPI	10.8	11.3	8.9	1.2
PCA	59.0	50.9	63.9	2.1
DPn	5.6	5.6	6.3	0.32
Dihydrochalcones				
PLX	0.87	0.94	0.68	0.02
PLZ	4.56	5.35	3.17	0.06
Hydroxycinnamic acids				
5CQA	17.42	22.26	17.39	0.23
pCQA	1.27	1.50	1.10	0.04
Flavonols				
TotalFl	1.74	1.82	0.67	0.22
Anthocyanins				
EC	nd	0.02	nd	0.00
CYA	nd	0.08	nd	0.00
**AIS ^2^**				
Rha	9	7	11	0.2
Fuc	5	4	1	0.2
Ara	76	71	81	5.7
Xyl	37	37	47	3.7
Man	15	12	9	3.3
Gal	49	45	45	4.0
Glc	214	174	254	11.9
GalA	148	144	170	4.4
MeOH (DM)	22 (80)	20 (78)	25 (81)	0.9 (4.6)

^1^ CAT: (+)-catechin, EPI: (−)-epicatechin, PCA: procyanidins, DPn: average degree of polymerization of procyanidins, PLX: phloretin-2-xyloglucoside, PLZ: phloridzin, 5CQA: 5-caffeoylquinic acid, pCQA: 4-*p*-coumaroylquinic acid quantified as *p*-coumaric acid, TotalFl: total flavonols quantified as quercetin, EC: (epi)catechin−cyanidin quantified as cyanidin, CYA: cyanidin. ^2^ AIS: alcohol insoluble solids, Rha: Rhamnose, Fuc: Fucose, Ara: arabinose, Xyl: xylose, Man: mannose, Gal: galactose, Glc: glucose, Gal A: galacturonic acid, MeOH: methanol, DM: degree of methylation. ^3^ Pooled SD: pooled standard deviation, *n* = 6, nd: not detected.

**Table 2:** Rates of SCFA production and procyanidin degradation during in vitro fermentation of the apple matrices with fecal microbiota (*n* = 3) from four healthy donors^1^.

Donor	Apple Matrices	Rate (h^−1^) ^1^			
		Acetate	Propionate	Butyrate	Procyanidins
O1	Mnc	0.131	0.081	0.066	0.171
	Mcov	0.207	0.105	0.105	0.043
	Mno	0.074	0.044	0.032	0.211
O2	Mnc	0.184	0.119	0.109	0.137
	Mcov	0.185	0.100	0.081	0.116
	Mno	0.115	0.075	0.065	0.191
O3	Mnc	0.116	0.126	0.070	0.188
	Mcov	0.158	0.168	0.063	0.126
	Mno	0.031	0.026	0.019	0.187
O4	Mnc	0.206	0.203	0.092	0.091
	Mcov	0.197	0.186	0.093	0.067
	Mno	0.100	0.115	0.048	0.125
	Pooled SD ^2^	0.027	0.032	0.019	0.027
***F*-value and significance**					
	Donor	3.560	4.598	1.820	2.452
		*	*	ns	ns
	Matrix	16.306	7.281	7.158	13.705
		***	**	**	***

^1^ Rate values were calculated according to Equation (1) (see Materials & Methods) and correspond to the mean of three independent measurements. ^2^ Pooled SD: pooled standard deviation. F-values and significance (* *p* ≤ 0.05, ** *p* ≤ 0.01, *** *p* ≤ 0.001) obtained from two way ANOVA (‘donor’ and ‘matrix’ effects); ns: not significant.

**Table 3:** Statistical analysis of the effect of matrix and donor on the production of microbial metabolites (SCFAs and phenolics) and degradation of procyanidin during in vitro fermentation of the apple matrices with fecal microbiota (*n* = 3) from four healthy donors ^1^.

Statistics	Acetate	Propionate	Butyrate	PCA	3,4-diOHPPA	3-OHPPA	4-OHPPA	3-PPA	3,4-diOHPAA	3-OHPAA	3,4-diOHPval	3-OHPval	3-OHPVA
F-value and significance													
Matrix	8.779	1.852	6.281	23.364	12.113	5.831	8.520	1.134	4.543	2.501	6.089	2.646	9.311
	**	ns	**	***	**	**	**	ns	*	ns	**	ns	**
Donor	3.138	9.067	3.329	0.404	4.224	3.579	15.977	18.129	13.607	2.259	41.739	11.098	34.339
	*	*	*	ns	*	*	***	***	***	ns	***	***	***

^1^ Fishers’ *F*-values and significance (* *p* ≤ 0.05, ** *p* ≤ 0.01, *** *p* ≤ 0.001) obtained from two way ANOVA (‘donor’ and ‘matrix’ effects) performed on microbial metabolites and residual procyanidins when they were at their maximal level of concentration or degradation during the kinetics of incubation of the apple matrices with the fecal microbiota, that is at 48 h for end-products and at 6, 8 or 10 h for intermediate products, as illustrated in Figure 1. PCA: procyanidins, 3,4-diOHPPA: 3-(3,4-dihydrophenyl)propionic acid, 3-OHPPA: 3-(3-hydrophenyl)propionic acid, 4-OHPPA: 3-(4-hydrophenyl)propionic acid, 3-PPA: 3-(phenyl)propionic acid, 3,4-diOHPAA: 3-(3,4-dihydrophenyl)acetic acid, 3-OHPAA: 3-(3-hydrophenyl)acetic acid, 3,4-diOHPval: 5-(3,4-dihydroxyphenyl)-ɣ-valerolactone, 3-OHPval: 5-(3-hydroxyphenyl)-ɣ-valerolactone, 3-OHPVA: 5-(3-hydroxyphenyl)valeric acid * (*p* ≤ 0.05), ** (*p* ≤ 0.01), *** (*p* ≤ 0.001).

## Supplementary Materials

The following are available online at https://www.mdpi.com/2072-6643/11/3/664/s1, **Figure S1**: Experimental design and samples used for microbiota analyses; **Figure S2**: Concentrations in mM of microbial metabolites in the fermenters at T0 and at T48 h after incubation of the 3 apple matrices with fecal microbiota (*n* = 3) from four healthy donors (O1 to O4). (a) Phenolic metabolites: 3,4-diOHPPA: 3-(3,4-dihydrophenyl)propionic acid, 4-OHPPA: 3-(4-hydrophenyl)propionic acid, 3-OHPPA: 3-(3-hydrophenyl)propionic acid, 3-PPA: 3-(phenyl)propionic acid, 3,4-diOHPAA: 3-(3,4-dihydrophenyl)acetic acid, 3-OHPAA: 3-(3-hydrophenyl)acetic acid, 3,4-diOHPval: 5-(3,4-dihydroxyphenyl)-ɣ-valerolactone, 3-OHPval: 5-(3-hydroxyphenyl)-ɣ-valerolactone, 3-OHPVA: 5-(3-hydroxyphenyl)valeric acid. (b) Short chain fatty acids. Each bar represents the mean ± standard deviation (*n* = 9). **Figure S3**: α-Diversity of bacterial communities based on amplified 16S rRNA gene sequences estimated by four different measures (observed OTU richness, Chao estimate of total richness, Shannon and Inverse Simpson diversity index). Diversity observed from fecal slurries according to experimental conditions i.e., at the initial time point of fermentation (Control) and after 48 h fermentation of the three apple matrices (Mcov, Mnc, Mno). (b) Diversity observed from fecal slurries according to subject for the four experimental conditions as in (a). Box centre lines show the medians, boxplot limits indicate the 25th and 75th percentiles, outliers are represented by dots. **Figure S4**: Proposed pathways for the catabolism of several phenolic compounds found in apple by human gut microbiota (Adapted from Aura, 2008 [12] and Del Rio et al., 2013 [55]). Chemical structures presented in blue are parent phenolic compounds while those in black correspond to their fecal microbial catabolites. The metabolite in frame reached a plateau after 48 h fermentation (end-product). The other ones were transitory metabolites. **Table S1**: Major ions of identified microbial metabolites (analyzed as their trimethylsilylated derivatives) detected during in vitro incubations of the apple matrices with the fecal microbiota from four healthy subjects, **Table S2**: Average abundances of OTUs in subjects O1, O2, O3, O4 (*n* = 3 per subject) expressed in read counts/1000 total reads. **Table S3**: Effect of the experimental conditions (Control, Mcov, Mnc, Mno) on the abundance of target bacterial groups estimated by qPCR.
