# Supplementary material for: Procyanidin—Cell Wall Interactions within Apple Matrices Decrease the Metabolization of Procyanidins by the Human Gut Microbiota and the Anti-Inflammatory Effect of the Resulting Microbial Metabolome In Vitro
Source: Nutrients. 2019 Mar 19;11(3):664. doi: 10.3390/nu11030664 (PMC6471247; doi:10.3390/nu11030664)
Supplement: Supplementary file 1 [file nutrients-11-00664-s001.zip › Supplementary Data_Nutrients-452796_Proofs.docx]

**Supplementary Data (Figures + Tables)**

**Figure S1.** Experimental design and samples used for microbiota analyses

**Figure S2.** Concentrations in mM of microbial metabolites in the fermenters at T0 and at T48 h after incubation of the 3 apple matrices with fecal microbiota (n=3) from four healthy donors (O1 to O4).

1. Phenolic metabolites: 3,4-diOHPPA: 3-(3,4-dihydrophenyl)propionic acid, 4-OHPPA: 3-(4-hydrophenyl)propionic acid, 3-OHPPA: 3-(3-hydrophenyl)propionic acid, 3-PPA: 3-(phenyl)propionic acid, 3,4-diOHPAA: 3-(3,4-dihydrophenyl)acetic acid, 3-OHPAA: 3-(3-hydrophenyl)acetic acid, 3,4-diOHPval: 5-(3,4-dihydroxyphenyl)-ɣ-valerolactone, 3-OHPval: 5-(3-hydroxyphenyl)-ɣ-valerolactone, 3-OHPVA: 5-(3-hydroxyphenyl)valeric acid. (b) Short chain fatty acids. Each bar represents the mean ± standard deviation (n= 9).

**Figure S3.** : α-Diversity of bacterial communities based on amplified 16S rRNA gene sequences estimated by four different measures (observed OTU richness, Chao estimate of total richness, Shannon and Inverse Simpson diversity index).

Diversity observed from fecal slurries according to experimental conditions *i.e.* at the initial time point of fermentation (Control) and after 48h fermentation of the three apple matrices (Mcov, Mnc, Mno). (b) Diversity observed from fecal slurries according to subject for the four experimental conditions as in (a). Box centre lines show the medians, boxplot limits indicate the 25th and 75th percentiles, outliers are represented by dots.

**Figure S4**. Proposed pathways for the catabolism of several phenolic compounds found in apple by human gut microbiota (Adapted from Aura, 2008 [12] and Del Rio et al., 2013 [55]).

Chemical structures presented in blue are parent phenolic compounds while those in black correspond to their fecal microbial catabolites. The metabolite in frame reached a plateau after 48h fermentation (end-product). The other ones were transitory metabolites.

**Figure S1**


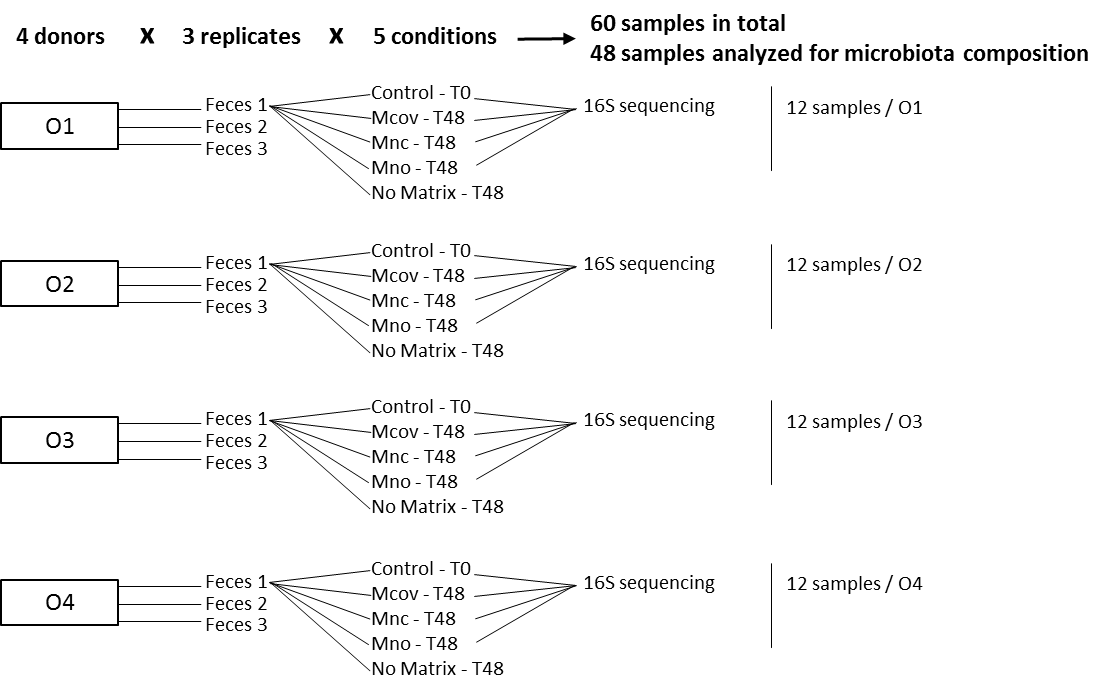


**Figure S2**

**(a)**

**
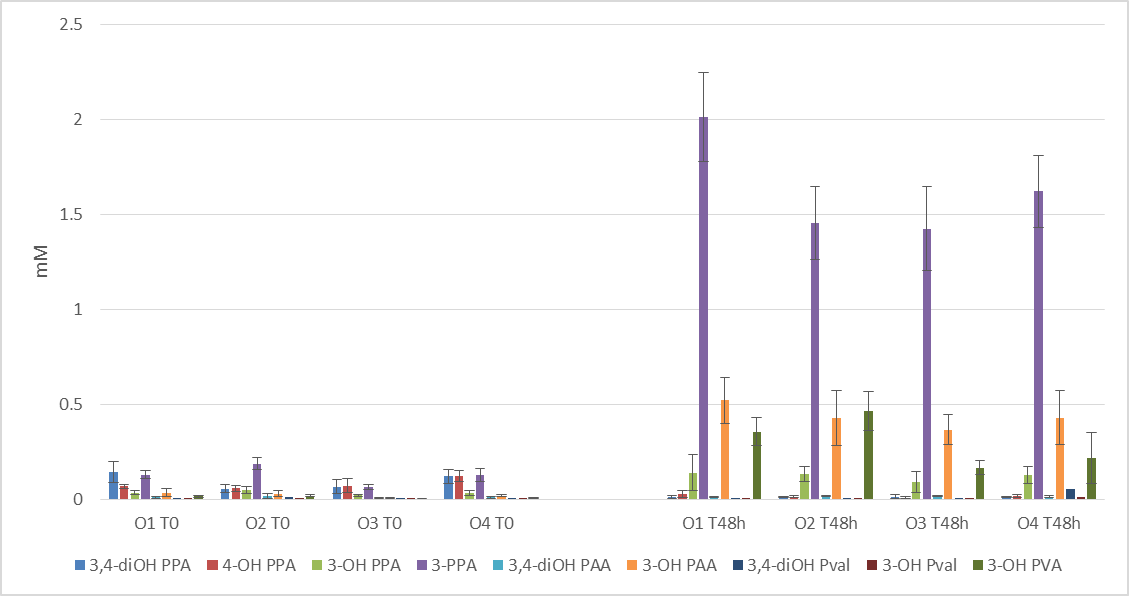
**

**(b)
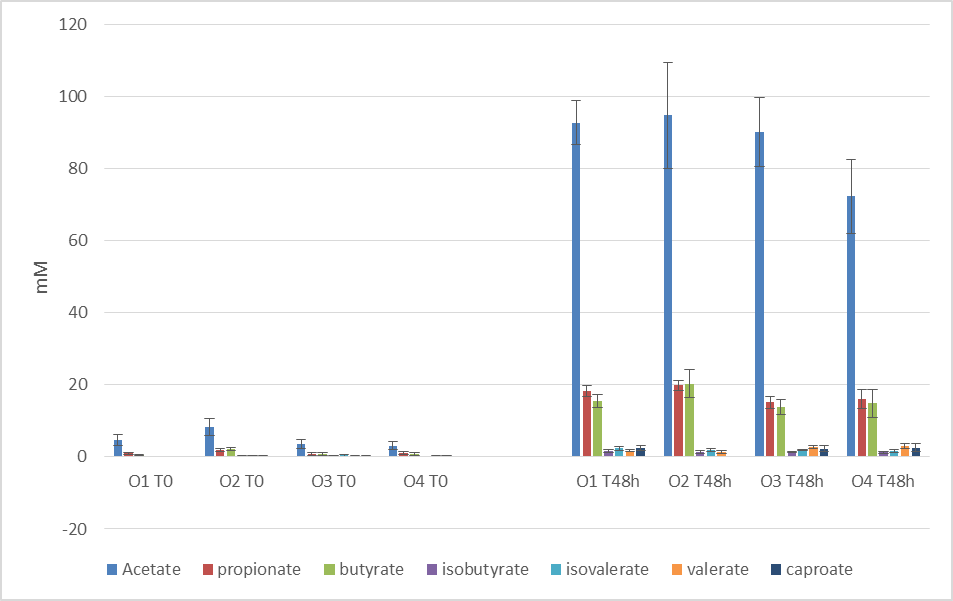
**

**Figure S3**


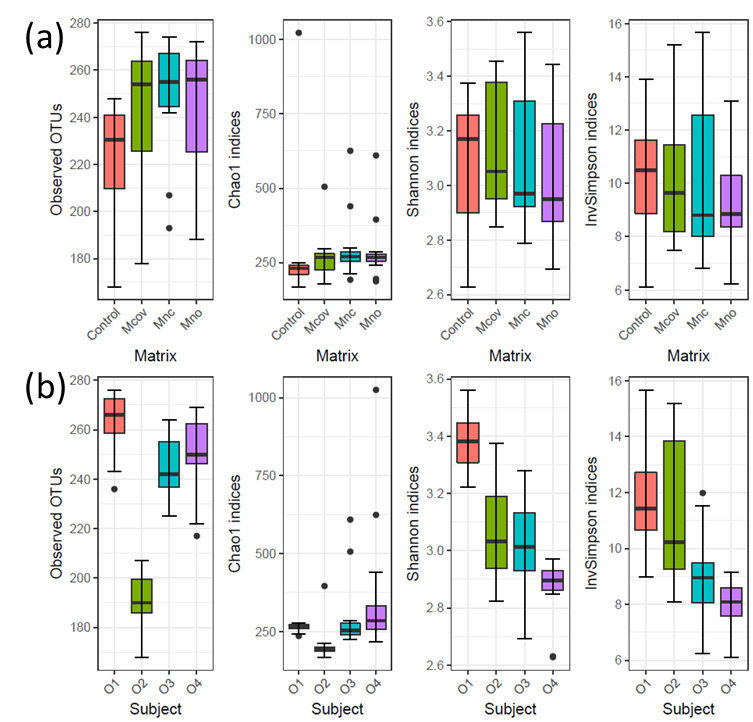


**Figure S4**

**
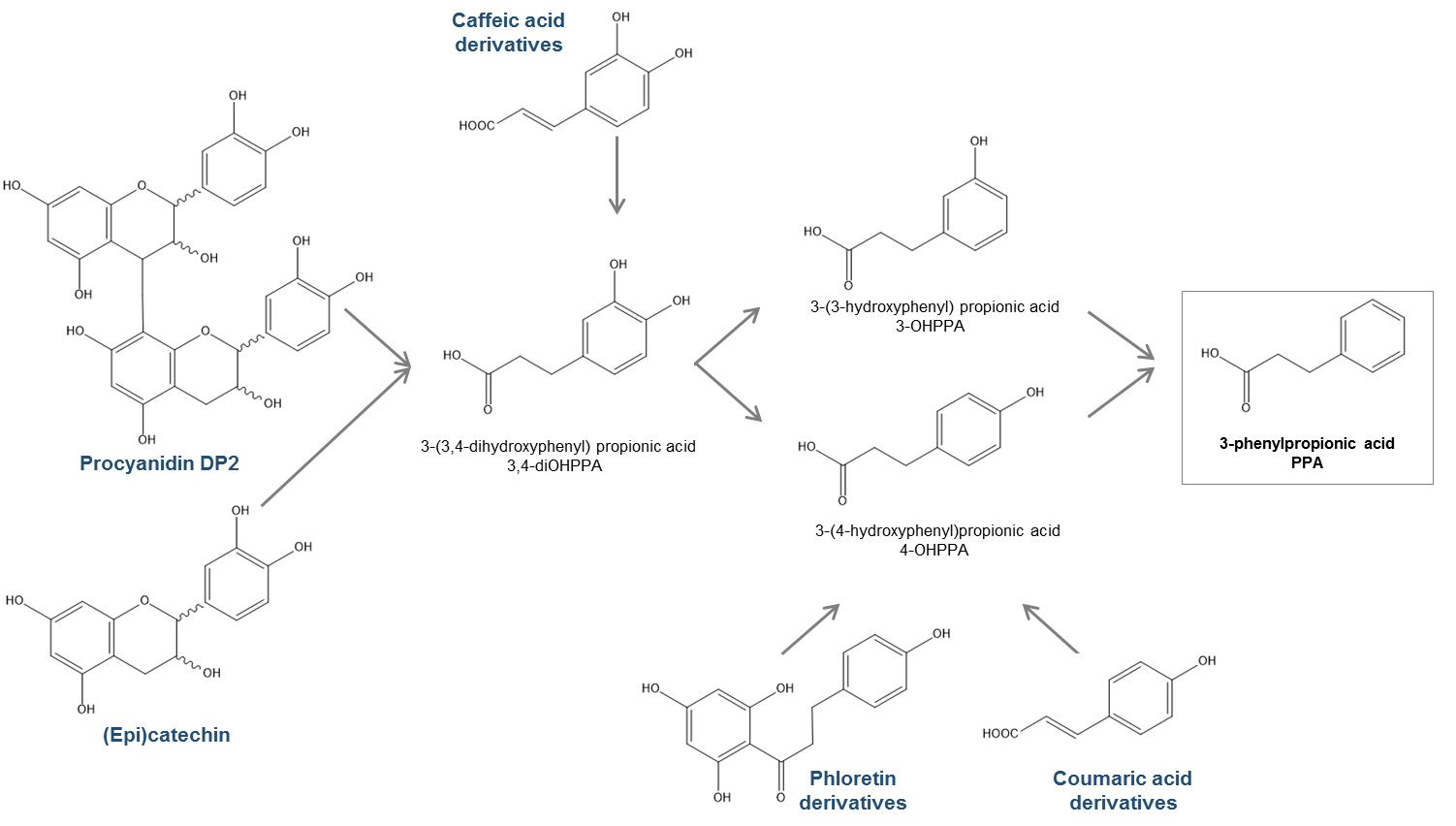
**

**Table S1.** Major ions of identified microbial metabolites (analyzed as their trimethylsilylated derivatives) detected during *in vitro* incubations of the apple matrices with the fecal microbiota from four healthy donors

| **Compounds** | **Mass** | **Main ions (relative intensity)** |
| --- | --- | --- |
| *Benzoic acids* |  |  |
| Benzoic Acid | 194 | 105(100), 179(90), 77(80), 135(50), 51(26), 45(16), 180(14), 73(9), 75(8), 43(8) |
| 3-hydroxybenzoic acid | 282 | 267(100), 73(58), 193(43), 282(37), 223(32), 268(22), 45(13), 126(10), 75(10), 283(9) |
| 4-hydroxylbenzoic acid | 282 | 73(100), 267(66), 223(41), 193(27), 282(20),45(17), 268(11), 126(11), 75(8), 74(6) |
| 3,4-dihydroxybenzoic acid | 370 | 73(100), 193(85), 370(38), 45(22) 355(22), 194(13), 371(13), 311 (10), 74(9), 281(8) |
| *Phenylacetic acids* |  |  |
| Phenylacetic acid | 208 | 73(100), 75(42), 164(17), 91(15), 193(15), 45(10), 74(10), 65(9), 165(5), 43(4) |
| 3-hydroxyphenylacetic acid | 296 | 73(100), 75(22), 164(14), 45(12), 147(11), 74(7), 281(6), 296(5), 40(4), 252(4) |
| 3,4-dihydroxyphenylacetic acid | 384 | 73(100), 384(46), 179(38), 267(30), 385(16), 75(16), 45(12), 369(9), 268(8), 74(8) |
| *Phenylpropionic acids* |  |  |
| 3-(phenyl)propionic acid | 222 | 104(100), 75(86), 73(51), 207(45), 91(26), 222(22), 105(13), 77(10), 45(9), 208(7) |
| 3-(3-hydroxyphenyl)propionic acid | 310 | 205(100), 192(81), 73(72), 75(60), 310(46), 193(36), 177(31), 45(19), 206(18), 179(15) |
| 3-(4-hydroxyphenyl)propionic acid | 310 | 179(100), 73(67), 192(64), 75(22), 310(21), 180(17), 177(15), 45(14), 55(13), 193(13) |
| 3-(3,4-dihydroxyphenyl)propionic acid | 398 | 73(100), 179(44), 398(20), 45(18), 75(15), 267(14), 74(9), 180(8), 399(7), 280(6) |
| *Phenylvaleric acids* |  |  |
| 3-(3-hydroxyphenyl)valeric acid | 338 | 73(100), 75(65), 180(38), 147(27), 338(19), 248(18), 323(16), 233(16), 206(15) |
| *Phenylvalerolactones* |  |  |
| 5-(3-hydroxyphenyl)-ɣ-valerolactone | 264 | 85(100), 264(34), 207(21), 179(20), 149(20) |
| 5-(3,4-dihydroxyphenyl)-ɣ- valerolactone | 352 | 267(100), 205(55), 179(53), 73(47), 352(45) |
| *Phenyllactic acids* |  |  |
| 3-phenyllactic acid | 310 | 193(100), 73(79), 147(76), 220(43), 194(41), 219(24), 295(22), 148(18), 267(17), 75(12) |
| 3-(4’-hydroxyphenyl)lactic acid | 398 | 73(100), 179(84), 147(24), 308(16), 45(14), 180(13), 74(9), 75(9), 47(7), 281(4) |

**Table S3. Effect of the experimental conditions (Control, Mcov, Mnc, Mno) on the abundance of target bacterial groups estimated by qPCR^1^**

|  | **Control T0** | **Mcov T48** | **Mnc T48** | **Mno T48** | **Std.Error** | **P value** |
| --- | --- | --- | --- | --- | --- | --- |
|  | Log10 rrs gene copies / µg extracted DNA | | | |  |  |
| ***Bacteroides*** | 7.24 | 7.46 | 7.26 | 7.35 | 0.35 | ns |
| ***Bifidobacteria*** | 7.21 | 7.29 | 7.55 | 7.33 | 0.43 | ns |
| ***Roseburia_E. rectale*** | 7.60 | 7.36 | 7.43 | 7.50 | 0.41 | ns |
| ***Faecalibacterium_prausnitzii*** | 7.82 | 7.80 | 7.87 | 7.58 | 0.32 | ns |

^1^The values correspond to the mean of quantifications performed with DNA extracted from fecal slurries (n=12, 4 subjects x 3 biological replicates) at the initial time point of fermentation (Control) and after 48h fermentation of the 3 apple matrices (Mcov, Mnc, Mno). ns: no significant
